# Assessing the Relative Sustainability of Point-of-Use Water Disinfection Technologies for Off-Grid Communities

**DOI:** 10.1021/acsenvironau.4c00017

**Published:** 2024-07-09

**Authors:** Bright C. Elijah, Ali Ahmad, Yalin Li, Jaime Plazas-Tuttle, Lewis S. Rowles

**Affiliations:** † Department of Civil Engineering and Construction, Georgia Southern University, Statesboro, Georgia 30458, United States; ‡ Department of Civil and Environmental Engineering, Rutgers, The State University of New Jersey, Piscataway, New Jersey 08854, United States; § Department of Civil and Environmental Engineering, Universidad de los Andes, Bogotá 111711, Colombia

**Keywords:** techno-economic analysis (TEA), life cycle assessment (LCA), drinking water, underserved communities, chlorination, ceramic water filter, UV disinfection

## Abstract

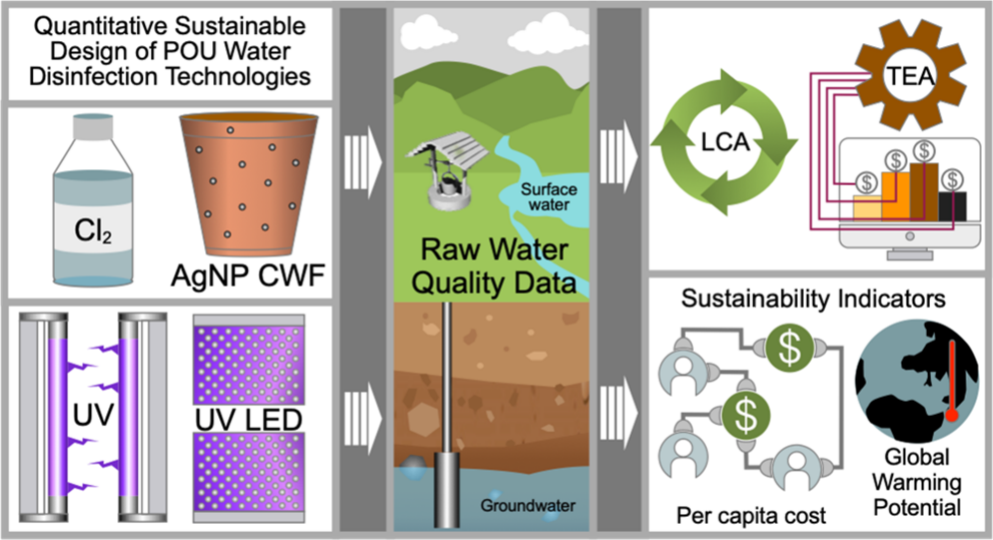

Point-of-use (POU) water disinfection technologies can be adopted to provide access to safe drinking water by treating water at the household level; however, navigating various POU disinfection technologies can be difficult. While numerous conventional POU devices exist, emerging technologies using novel materials or advanced processes have been under development and claim to be of lower cost with higher treatment capacity. However, it is unclear if these claims are substantiated and how novel technologies compare to conventional ones in terms of cost and environmental impacts when providing the same service (i.e., achieving a necessary level of disinfection for safe drinking water). This research assessed the sustainability of four different POU technologies (chlorination using sodium hypochlorite, a silver-nanoparticle-enabled ceramic water filter, ultraviolet mercury lamps, and ultraviolet light-emitting diodes). Leveraging open-source Python packages (QSDsan and EXPOsan), the cost and environmental impacts of these POU technologies were assessed using techno-economic analysis and life cycle assessment as per capita cost (USD·cap^–1^·yr^–1^) and global warming potential (kg CO_2_ eq·cap^–1^·yr^–1^). Impacts of water quality parameters (e.g., turbidity, hardness) were quantified for both surface water and groundwater, and uncertainty and sensitivity analyses were used to identify which assumptions influence outcomes. All technologies were further evaluated across ranges of adoption times, and contextual analysis was performed to evaluate the implications of technology deployment across the world. Results of this study can potentially provide valuable insights for decision-makers, nonprofit organizations, and future researchers in developing sustainable approaches for ensuring access to safe drinking water through POU technologies.

## 1 Introduction

The United Nations has established a set of Sustainable Development Goals (SDGs) as part of their global agenda to address various social, economic, and environmental challenges. The SDG 6 is centered around universal access to safe and affordable drinking water by 2030, with a focus on the 2.1 billion people that lack access to safely managed water globally.^1^ One of the primary issues with poor water quality is microbial contamination which can cause potential acute health hazards, e.g., gastrointestinal infections, waterborne diseases, respiratory infections, etc.^2,3^ To supply safe and potable drinking water, centralized treatment facilities typically remove pathogens through both physical and chemical methods. While such facilities are common in developed countries, centralized systems are costly and require extended construction periods, especially when considering distribution systems.^4^ For example, a new water distribution system in lower-income countries is estimated to cost 64–268 USD·person^–1^ for 500–2000 households.^5^ Another estimation for the implementation of a piped water supply for a small town in Ghana is in the range of 10–14 USD·person^–1^·yr^–1^ (national minimum wage is approximately $689 USD·yr^–1^).^6^ It is notable that these estimated costs are only for the distribution system and do not include the cost of water treatment. These barriers make potable piped water out of reach for many developing countries or emerging economies, where the need to disinfect water is urgent. As an alternative solution to centralized systems, water can be disinfected at the household level or point of use (POU), providing a potential pathway for immediate safe drinking water for off-the-grid communities.^7^

Numerous POU disinfection technologies are commercially available, ranging from conventional technologies (e.g., boiling and POU chlorination) to new technologies (e.g., ultraviolet (UV) disinfection systems).^8^ For example, solar water disinfection (SODIS) can be a relatively simple intervention for disinfection when properly utilized. A year-long study in Cameroon highlighted that SODIS provided up to a 42.5% reduction in the risk for diarrheal diseases in households that properly treated their water, but only 45.8% of all households effectively adhered to the recommended practices of SODIS.^9^ Ceramic water filters are another POU technology that can be produced with local materials and provide dual mechanisms to remove bacteria, i.e., the porous physical ceramic matrix filtration and silver nanoparticle antimicrobial coating.^10,11^ A randomized controlled field trial in Bolivia demonstrated the effectiveness of ceramic filters in meeting the World Health Organization (WHO) drinking water standards. While results obtained proved valuable in achieving compliance when faced with turbid challenge waters, additional research is needed. One aspect that requires attention is the maintenance of ceramic filters, as an agent-based model has shown that neglecting it can hinder their long-term sustainability, despite their relative ease of use.^12,13^ Overall, sustained adoption of individual POU technologies can vary between communities due to contextual and end-user factors, but inadequate clarity on how decision-makers and stakeholders can navigate the different POU technologies under different contexts can limit implementation and sustained adoption. Therefore, the sustainability of numerous POU technologies needs to be simultaneously assessed while considering context-specific factors that enable engineers, agencies, and researchers to make informed decisions and select the most suitable treatment technology for a specific community.

Toward this end, techno-economic analysis (TEA) and life cycle assessment (LCA) can serve as valuable methods for evaluating trade-offs in terms of cost and environmental impacts when comparing different POU technologies. For instance, a study conducted on POU chlorination (Aquatabs), flocculant disinfection (Procter and Gamble Purifier of Water), and ceramic filters evaluated the cost-effectiveness considering costs related to startup, management, and logistics.^14^ While POU chlorination was found to be the most cost-effective method, this study was limited to a 1 year period, which may be relatively shorter than necessary in other contexts. A recent LCA of four UV-based systems, chlorination, and trucked water delivery found chlorination to have the lowest environmental impacts over various time and scale horizons.^15^ Leveraging both TEA and LCA can help identify trade-offs between cost and environmental impacts for POU technologies. These tools together have been used in a limited way to evaluate several conventional disinfection technologies (boiling, ceramic filters, biosand filters, and POU chlorination). Under a specific set of assumptions, boiling and chlorination had the highest environmental impacts, while boiling was the most expensive (0.053 USD·L^–1^) and chlorination was the least expensive (0.0005 USD·L^–1^).^16^ In general, accurately comparing the relative sustainability among different studies can be difficult due to variations in assumptions, leading to different outcomes for sustainability indicators. For example, shorter studies with technology lifespans of less than 1 year may not consider all materials and supplies that are used in the process throughout the technology’s lifetime. Considering the inherent uncertainty associated with changes over the lifetime, location, and other factors while assessing the relative sustainability of POU technologies can help to account for the fluctuating assumptions.

The goal of this study is to assess the relative sustainability of several readily available POU disinfection technologies. Specifically, the objectives of this work are to (i) characterize the overall cost and environmental impacts while considering necessary disinfection efficacy of these technologies and (ii) elucidate drivers for sustainability to better inform appropriate adoption in specific contexts. The technologies assessed in this study include POU chlorination, silver nanoparticle-enabled ceramic water filter (AgNP CWF), UV with a mercury lamp, and UV with a light-emitting diode (LED). This study leverages the quantitative sustainable design (QSD) methodology^17^ for TEA, LCA, and disinfection efficacy assessment using an open-source Python packages QSDsan (QSD for sanitation and resource recovery systems).^18,19^ Uncertainty was incorporated in the assumptions inputted into the models, and sensitivity analysis (via Spearman’s rank correlation coefficients) was completed to identify key drivers of sustainability. The impact of water quality was evaluated by updating assumptions considering two different water compositions (surface and groundwater), and a technology adoption period ranging from 1 to 15 years was assessed. A contextual analysis was also included to reflect the implications of location-specific parameters on technology deployment in 10 different communities across the world. The findings of this study are expected to offer valuable insights for decision-makers, nonprofit organizations, and future research endeavors focusing on sustainable approaches to safe drinking water through POU technologies.

## 2 Methodology

### 2.1 POU Disinfection Technologies

To explore trade-offs among POU technologies, we leverage the QSD methodology^17^ and software QSDsan,^20^ where integrated TEA and LCA were performed with parameters covering design, materials, energy and capital requirements, and operation and maintenance requirements (Figure S1). All essential decision variables and technological parameters were derived from a comprehensive range of sources (published research, manufacturers’ specifications, and guideline reports). All of the Python scripts are publicly available on GitHub with a README file for instructions,^21^ and an online (i.e., without local installation of Python) Python environment capable of running the scripts in Jupyter Notebook can be accessed in web browsers through the binder link on QSDsan GitHub repository.^22^ All input assumptions are included in the Supporting Information (SI).^22^ A 5 year technology adoption period and an average household size of 4 people were used as the baseline for all of the POU technologies. The number of people per household is aligned with the average number of households in most of the countries with lower access to basic drinking water.^23,24^ Two types of raw water (surface water and groundwater) were modeled with their characteristic water quality parameters (described in detail below). To standardize the disinfection efficacy of the technologies, a minimum of 3 log_10_ reduction was evaluated for all systems. It is notable that some POU technologies may inactivate microbes rather than fully disinfecting the water. Inactivation renders the microbes unable to reproduce and cause infection, while disinfection destroys or removes the microbes from the water. Since mechanistic removal pathways of pathogens are beyond the scope of this work, we use the terminology disinfection throughout.

#### 2.1.1 POU Chlorination

The disinfection method for POU chlorination was designed based on the use of a solution of sodium hypochlorite (NaClO). This solution is used to disinfect drinking water in households with a relatively simple setup (Figure S2). The specific NaClO product used here is marketed as WaterGuard, and each bottle contains 150 mL of the NaClO solution.^25^ The treated water volume was 20 L based on the assumed container capacity. This disinfection method is designed to be relatively simple to use, where the bottle cover of the WaterGuard bottle is used to dose the NaClO solution into 20 L of raw water. An expected one WaterGuard bottle cap is a measure of a single dose of NaClO solution, while two are used for a double dose. The full materials and cost inventory data for POU chlorination system are accounted for (Table S1). The code of this system was designed for three influent streams, i.e., the raw water, NaClO (chlorine stream), and polyethylene (WaterGuard bottles). To keep the goal of the minimum log_10_ reduction of bacteria, the dosing of NaClO was 1.88 mg·L^–1^ at low turbidity (≤10 NTU), and a double dose of 3.75 mg·L^–1^ was used at higher turbidity (>10 NTU).^26^ Algorithms were developed to capture this impact of water quality on cost and environmental impacts.

#### 2.1.2 AgNP CWF

The CWF is coated with AgNPs such that the ceramic matrix filters for a combination of physical (through filtering) and chemical (through AgNPs) disinfection.^10−12,27^ As shown in Figure S3, the setup has the ceramic coated with AgNP placed over a plastic bucket that holds the filtered and treated water. Materials used to make the setup (sawdust, clay, wood for the filters, and polyethylene for the plastic container) were incorporated to account for their costs and environmental impacts (Table S2).^13^ Additionally, the embedded electricity and propane to produce the filters are accounted for as capital requirements. The unit has one influent stream of raw water and an effluent of treated water. Here, AgNP is the main consumable as recoating will be needed after every 0.5 to 4.5 years. Algorithms in this unit were developed to account for the length of time before recoating the filter with AgNPs was necessary based on the quality of the water type. The lifetime of AgNPs in this unit depends on the water quality. Specifically, more frequent recoating is expected for higher turbidity and hardness because these constituents have been reported to remove more AgNPs.^10,11^ In this analysis, turbidity >10 NTU and/or hardness >60 mg CaCO_3_·L^–1^ were used as the thresholds for more frequent recoating of AgNPs.

#### 2.1.3 UV with Mercury Lamps

Low-pressure mercury lamps were used to provide UV radiation for bacteria disinfection at a wavelength between 200 and 280 nm.^26^ The system in this study has two UV lamps on opposite sides with water flowing through a quartz tube to maximize the light transmittance to microbes (Figure S4). The materials accounted for included lamps, aluminum, polyethylene, and polyvinyl chloride (Table S3).^14^ The mercury UV lamps used in this work were expected to have a lifespan of approximately 2000 h; however, varying lifespan has been reported by manufacturers of other mercury lamps.^15^ The unit is modeled to have two mercury lamps that use 30 W of electricity each. It is designed based on a flow of 9.46 L·min^–1^ with a UV dose of 187 mJ·cm^–2^, both with uncertainty ranges based on the literature for *Escherichia coli* disinfection.^14^ In this unit, we incorporated the impact of water quality through turbidity on UV light transmittance, UV dose, and detention time. These factors influence the energy requirements and potential cost and environmental impacts. The UV lamp was assumed to be on for double the time in higher turbidity (>10 NTU) to account for the increased retention time in water with less UV light transmittance. The extended residence time was also accounted for in the unit’s electricity demand. Specifically, the electricity use of the UV mercury lamp system was modeled based on the duration during which the lamps were operated. While this approach captures the impact of water quality on energy use, it does not fully account for variations in energy requirements with different UV doses. This simplifying assumption was necessary due to limited data. Future work could refine the energy model by incorporating dose-dependent energy functions if additional data becomes available.

#### 2.1.4 UV with LEDs

The last POU technology in this study is UV with LEDs as the source of disinfection. These lights generally are considered more environmentally sustainable as they do not contain mercury like the lamps for traditional UV systems.^14^ This unit also allows the UV dose to be adjusted and offers design flexibility, as the UV LEDs can be arranged in different formats to optimize disinfection (Table S4). The design capital materials included quartz, stainless steel, aluminum, and 30 UV LEDs. The unit was designed in a flow-through system so the water is surrounded by arrays of UV LEDs separated by a quartz material that allows adequate transmittance of UV lights for disinfection. As shown in Figure S5, the plan view UV LEDs are set up with 15 LEDs on each side of the unit.^16^ The system is set up such that an array of UV LEDs require 23 W of electricity.^28^ It is designed based on a flow of 0.19 L·min^–1^ with a UV dose of 187 mJ·cm^–2^, both with uncertainty ranges based on the literature for *E. coli* disinfection.^29^ UV LEDs used in this study are estimated to have a lifespan of approximately 10,000 h. Other studies and manufacturers have reported higher lifetime of up to 100,000 h, although many of these are still in the developing stage.^29^ The unit is designed to incorporate the influence of water quality similar to the unit for UV with a mercury lamp. Turbidity of >10 NTU was assigned a double retention time factor, which was used for the accounting of electricity demand and the lifetime of the lamps. Similar to the UV mercury lamp system, the electricity use of the UV LED system was modeled based on the operating time of the LEDs. The LED operation time was doubled for higher turbidity water to account for the longer detention time needed to deliver the target UV dose. While this approach captures the key impact of turbidity on energy use, it does not fully account for dose-dependent variations in energy requirements. This simplifying assumption was necessary due to limited data.

### 2.2 Water Quality and Disinfection Efficacy

In order to account for the effect of water quality, surface water and groundwater were modeled based on parameters and assumptions derived from the literature. *E. coli* was selected as the indicator microbe to evaluate the level of contamination in the study. The algorithms for the operation and maintenance requirements for each technology were designed to achieve a consistent log_10_ reduction and disinfection efficacy of 3 logs (at a minimum) for all systems evaluated.^30^ Specifically, the costs and environmental impacts were normalized using the functional unit of water needed per capita-year with 3 log_10_ removal of bacteria. To achieve the set level of efficacy for each system, a comprehensive assessment was conducted to determine the necessary capital materials and consumables based on raw water quality. This assessment had implications for both cost and environmental considerations, as shown in the system boundaries and foreground inventory for the four point-of-use disinfection systems (Figure S6).

To capture the key dependencies between design and process parameters, the system algorithms link relevant parameters, even though their uncertainty ranges are modeled independently. For example, turbidity levels influence the UV transmittance, dose, and detention time in the UV system algorithms to account for the impact on the indicators of cost and environmental impacts. The uncertainty in each of those parameters is modeled independently since sufficient data were not available to quantitatively model all correlations. However, the algorithms still capture the functional relationships between the parameters for a given Monte Carlo simulation run. Similar dependencies are included for the other POU systems as well, such as the impact of turbidity on the chlorine dose and AgNP recoating frequency. Future work could incorporate quantitative models of parameter correlations if additional data becomes available.

Therefore, the different water quality parameters for groundwater and surface water sources serve as the contextual parameters modeled into the systems. For instance, groundwater source will most likely have higher hardness due to water dissolving minerals as it moves through rocks.^31^ Both waters have 1500–2500 CFU·100 mL^–1^ of *E. coli*.^11^ A characteristic groundwater had a turbidity of 1–10 NTU and hardness of 60–120 mg·L^–1^ as CaCO_3_, and characteristic surface water had a turbidity of 10–30 NTU and hardness of 0–60 mg·L^–1^ as CaCO_3_ (Table S5).^31^ The same *E. coli* concentration was selected for both waters to help focus the study on navigating the impact of the other water quality parameters. It is notable that microbial concentration can vary greatly between waters and even temporally within the same water source. *E. coli* was selected due to being an indicator microbe that has been widely studied for disinfection; thus, numerous lab and field studies on the POU technologies could be integrated in this study for comparison of costs and environmental impacts.

### 2.3 Economic Analysis

TEA was leveraged to assess the economic requirements of each POU technology. We accounted for capital, operation, and maintenance costs and energy costs. All costs were normalized to the economic indicator of USD·cap^–1^·yr^–1^. To assess the cost-effectiveness of the POU technologies over different time periods, the capital and operating costs were annualized over the expected lifetime of each system. These annualized costs represent the average yearly costs of owning and operating the POU technologies, assuming that the costs are evenly distributed over the lifespan. It is important to note that the actual cash flows in each year could vary, with higher costs in the initial year of purchase and in years when major components need replacement. Specifically, capital cost covered all of the purchases that the units required at start (e.g., housing for the UV systems, water storage bottles), while operation and maintenance accounted for cost estimates of all consumables materials and parts that require periodic replacements (e.g., NaClO, lamps, AgNP coating). Energy cost requirements were accounted for, depending on the electricity need of each unit. It is notable that this requirement does not apply to units without electricity use (i.e., POU chlorination and AgNP CWFs). Energy was accounted for separately from operation and maintenance to better understand its individual contribution to financial sustainability. The initial step of TEA involves identifying the specific objective for cost assessment, determining the components comprising the technology, and identifying the various factors that contribute to the overall cost (e.g., cost of UV lamp and labor cost). The next step entails data compilation of the cost associated with each material and determination of the frequency at which such costs will be applicable in cases involving replaceable parts. It is important to note that capital costs are also spread out through the analysis period (5 years of baseline period). Discounted cash flow analysis was applied to account for future value of money over the technology’s lifespan with a 5% discount rate on average.^32^ Subsequently, the following step involves identifying and considering capital costs associated with construction, operation, and maintenance over the entire duration of the analysis. The cost analysis was designed to account for the impacts of water quality from each unit while achieving necessary disinfection efficacy. Salvage costs were not accounted for in this analysis.

### 2.4 Environmental Analysis

LCA of the POU technologies encompassed impacts from capital inputs, operational activities, maintenance requirements, and energy consumption. Life cycle greenhouse gas emission impact data was obtained from EcoInvent v3.9 database considering all materials and consumables in each unit, and global warming potential (GWP) was selected as the environmental sustainability indicator through the U.S. EPA’s TRACI (Tool for the Reduction and Assessment of Chemicals and Other Environmental Impacts) method (Table S6).^33,34^ The LCA methodology employed in this study followed several key steps. First, the goal and scope were established to track the environmental impacts associated with both the capital inputs and the operation and maintenance requirements of the analyzed POU technologies. The inventory analysis was used to account for all of the materials and their respective weights (in kg) and other relevant parameters (such as the number of UV lamps utilized) in each POU system.^20^ The impact assessment phase incorporated the GWP for the identified parameters and materials. It is notable that energy was accounted for separately from operation and maintenance to better understand its individual contribution to environmental sustainability.

### 2.5 Uncertainty and Sensitivity Analyses

Uncertainty was incorporated into all assumptions and data for each parameter by introducing a range of 5–25% of uncertainty distribution depending on the data availability and level of confidence. For example, higher uncertainty (up to ±25%) was used when less data was available. Uncertainty was added to an expected value with uniform distributions used by default unless additional data was available. Some variables were set to be constant based on their meaning (e.g., number of lamps in a UV system). The details on the assumed uncertainty ranges and distributions are included in Tables S1–S6. The incorporated uncertainties capture variation in the values for all of the data points, e.g., fluctuation in materials cost and impacts. To address and quantify uncertainty, a total of 10,000 Monte Carlo simulations were conducted.^35^ Sensitivity analysis was performed to determine factors and parameters that are key drivers to changes in system’s cost and environmental impacts. Specifically, we used Spearman’s rank correlation coefficients to measure and analyze the sensitivity of individual parameters for all units to cost and environmental impacts.^17^ Here, we report the absolute value for the top five Spearman’s rank correlation coefficients (>|0.05| and *p*-value <0.05) for total cost and GWP for each technology in each water.

### 2.6 Impact of Technology Adoption Lifetime

The baseline assumption in this study was that each POU technology would be utilized for a duration of 5 years. However, to gain deeper insights, the analysis further examined the impact of adopting POU technologies for different lifetimes. Depending on the context, these technologies may be deployed for a relatively short period (e.g., after extreme weather events cause interruption of a centralized water supply) or a longer period (e.g., as a primary treatment method in underserved communities). The performance of each technology was simulated by setting the usage period to 1, 2, 5, 10, and 15 years. The design and process algorithms for each technology were adjusted accordingly to account for the change in the usage period to obtain the net cost and net GWP associated with different lengths of technology adoption.

### 2.7 Contextual Analysis

To provide insight into deploying the four POU technologies across the world, a contextual analysis was performed to assess the implications of contextual parameters specific to the deployment site. Demographic (household size), water quality (*E. coli*, turbidity, hardness), and energy (electricity cost and GWP characterization factor) data were collected from 10 different communities. These communities include two from Africa (Kampala, Uganda;^36,37^ Limpopo, South Africa^38^), two from Asia (Gunungkidul, Indonesia;^39^ Panobolon Island, Philippines^40^), four from North America (Colonias, United States;^41,42^ Navajo Nation, United States;^11^ Les Anglais, Haiti;^43^ Oaxaca, Mexico^44^), and two from South America (Santa Cruz, Bolivia;^45^ Antioquia, Colombia^46^). The collected data were then used in TEA and LCA to obtain location-specific cost and GWP (Table S7).

## 3 Results and Discussion

### 3.1 Economic and Environmental Sustainability of POU Technologies in Varying Water Quality

#### 3.1.1 Techno-Economic Analysis

**Figure 1:**
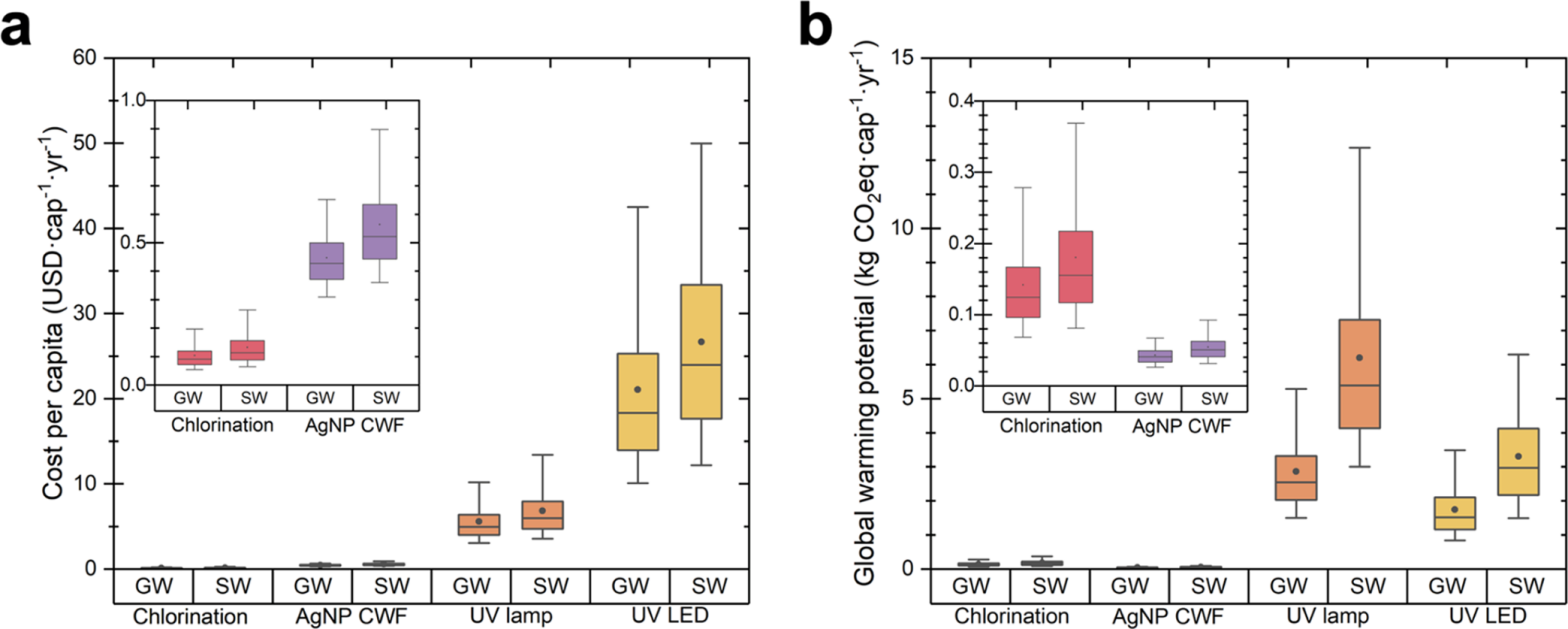
Estimated costs (a) and global warming potential (b) of POU technologies for groundwater (GW) and surface water (SW). Costs and impacts are annualized over the lifetime of the systems. The plots show the cost and environmental impacts on the ordinate and the POU technologies on the abscissa. Boxes and whiskers show the median values (center line), 25th and 75th percentiles (bottom and top of the box), 5th and 95th percentiles (lower and upper whiskers), and means (point) from the uncertainty analysis of 10,000 Monte Carlo simulations. These results assume a baseline adoption period of 5 years.

For the groundwater, the POU chlorination system was found to have the lowest cost with a net cost of 0.09 [0.05–0.020] USD·cap^–1^·yr^–1^ (median [5th percentile −95th percentile hereinafter]; Figure 1a). The next lowest cost was AgNP CWF at 0.43 [0.31–0.65] USD·cap^–1^·yr^–1^. UV (mercury) lamp had a net cost of 4.96 [3.04–10.18] USD·cap^–1^·yr^–1^, and the highest net cost was for UV LED which was 18.32 [10.08–42.49] USD·cap^–1^·yr^–1^. The cost-effectiveness of POU chlorine treatment can be attributed to the utilization of simple and affordable materials like 20 L jerrycans and WaterGuard (NaClO) bottles, which are available at a cost ranging from 0.08 to 0.33 USD per bottle.^47^ The low cost of AgNP CWF can be attributed to the low cost of capital materials and production, along with low-cost requirements for operation and maintenance. The only consumable for AgNP CWFs is the AgNP recoating, which is not as frequent compared to the POU chlorination system that relies strictly on more affordable consumable NaClO. It is notable that recoating of AgNP would require trained individuals. In contrast, UV systems employing mercury lamps and UV LEDs involve relatively higher costs due to the requirement for more expensive materials and the consumption of electricity during operation. When the two UV systems are compared, it is observed that UV LEDs are generally more expensive than mercury lamps. However, UV LEDs offer a longer lifespan and have lower electricity requirements compared with traditional mercury lamps.

In the case of surface water, the net cost followed the same order of groundwater, but the specific cost estimates were higher for each technology. The net cost for surface water, from lowest to highest, was as follows: POU chlorination (0.11 [0.07–3.65] USD·cap^–1^·yr^–1^), AgNP CWF (0.52 [0.36–0.90] USD·cap^–1^·yr^–1^), POU UV mercury lamp (5.96 [3.57–13.40] USD·cap^–1^·yr^–1^), and UV LED (23.97 [12.19–49.97] USD·cap^–1^·yr^–1^). The higher operation and maintenance cost associated with surface water is primarily due to the need for replaceable parts or consumables. This cost increase is more significant for chlorination and is only slightly higher for AgNP CWFs. Specifically, due to the higher turbidity level, the surface water required a higher dose (doubling the dose) of NaClO for POU chlorination.^25^ Similarly, the increase in turbidity required more frequent recoating for the AgNP CWFs. It is worth noting that the increase in the cost for the AgNP CWF is marginal. The turbidity in surface water also leads to an increased electricity run time for UV mercury lamps and UV LEDs, resulting in higher electricity costs and more frequent lamp replacements. However, these additional costs have a minimal impact on the overall costs of the UV mercury lamps and UV LEDs. Overall, the higher operation and maintenance costs associated with surface water result in higher net costs for deploying POU technologies when treating raw water with similar water characteristics to groundwater.

#### 3.1.2 Life Cycle Assessment

Regarding environmental impacts, for groundwater, AgNP CWF technology exhibited the lowest overall GWP, estimated to be 0.04 [0.03–0.07] kg of CO_2_ eq·cap^–1^·yr^–1^, which was followed by POU chlorination with an estimated GWP of 0.12 [0.07–0.28] kg of CO_2_ eq·cap^–1^·yr^–1^ (Figure 1b). The UV LED had a higher GWP of 1.51 [0.84–3.49] kg CO_2_ eq·cap^–1^·yr^–1^, while the UV mercury lamp technology had the highest GWP, estimated at 2.55 [1.50–5.29] kg CO_2_ eq·cap^–1^·yr^–1^. For both UV systems, the impact on GWP from capital materials was greater than that from operation and maintenance, which mainly consisted of electricity consumption and lamp replacement. However, the POU chlorination system was also influenced by operation and maintenance costs due to the need for consumable NaClO.

For the surface water, the estimated GWP ranges from the lowest to highest as follows: AgNP CWF (0.05 [0.03–0.09] kg CO_2_ eq·cap^–1^·yr^–1^), POU chlorination (0.16 [0.08–0.37] kg CO_2_ eq·cap^–1^·yr^–1^), UV LED (2.97 [1.49–6.30] kg CO_2_ eq·cap^–1^·yr^–1^), and UV mercury lamp (5.39 [3.00–12.37] kg CO_2_ eq·cap^–1^·yr^–1^). The GWP estimates show a similar order of impact from the lowest to highest impact for both water types. However, due to the impact of water quality on materials requirements (such as NaClO dosage, AgNP recoating, and lamp lifetime), the GWP associated with surface water is relatively higher for all POU technologies compared to groundwater. These results align with the trends observed in the TEA of surface water.

Overall, the cost and environmental impacts of these POU disinfection technologies can be directly influenced by the water quality. Turbidity in treated water necessitates increased consumables for effective disinfection across all technologies. These consumables can have a direct influence on the overall sustainability. Understanding the capital, operation, and maintenance requirements can help inform the deployment of these POU technologies in various contexts. For instance, chlorination relies heavily on the NaClO supply chain, while UV systems require a readily available electricity source. While the overall finding of chlorination and AgNP CWFs being affordable POU technologies has been reported in the literature, the findings here give a more in-depth and nuanced level of TEA.^30^ This level of analysis reveals that characteristics of the source water can significantly impact sustainability, and the specific requirements of each technology offer different opportunities for deployment.

### 3.2 Elucidating Drivers of Sustainability

#### 3.2.1 Elucidating Drivers for Net Cost

**Figure 2:**
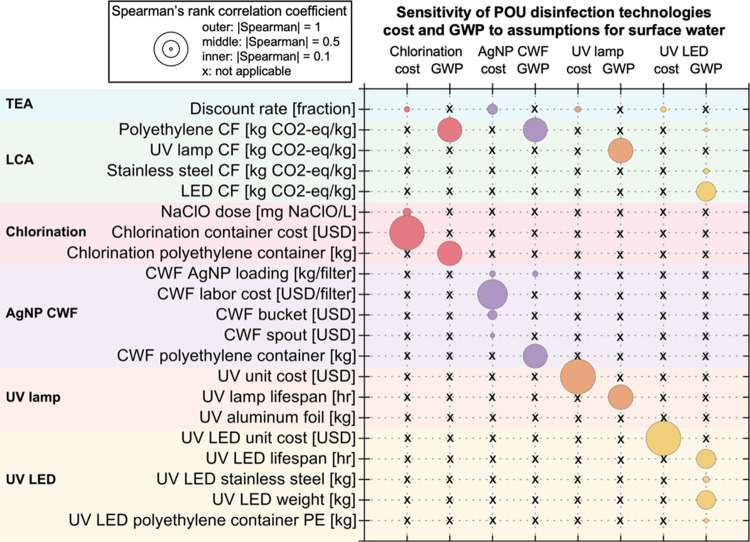
Spearman’s rank correlation for net cost and GWP for all POU technologies with surface water. The key drivers are on the ordinate corresponding with each technology’s cost and GWP on the abscissa. These results assume a baseline adoption period of 5 years.

Overall, the key drivers were similar for the disinfection of groundwater and surface water (Figures 2 and S7). The discount rate was found to have a noticeable influence on the cost of all four of the technologies. For POU chlorination, the assumptions that influenced cost were the dose of NaClO and the chlorination container cost (Figure 2). This outcome is expected since NaClO is the primary consumable in this technology, and the container is the only capital requirement. In the case of AgNP CWF, the key drivers were labor cost, bucket cost, and spout cost. With the AgNP CWF, the key drivers were AgNP loading rate, labor cost, discount rate, bucket cost, and lid cost. Notably, the labor cost had the greatest impact on the cost of AgNP CWF for both water types. While most of the key drivers for AgNP CWF were related to capital expenses, the AgNP loading rate was a key driver because of the required recoating to ensure proper disinfection efficacy. Regarding the two UV-based systems, unit cost was a common driver for both. These UV systems are inherently a more expensive option compared to chlorination and AgNP CWF. However, it is notable that the electricity cost did not significantly influence the cost of the UV systems.

#### 3.2.2 Elucidating Drivers for GWP

The key drivers of GWP for each technology are presented concerning the two water types (Figures 2 and S7). For POU chlorination, the key drivers of GWP were the weight of the polyethylene (PE) container and the PE characterization factor. The 20 L plastic container used in the system had a significant impact on the LCA of the POU chlorination system for both water types as it is a capital component of the system.

Regarding AgNP CWF, the key drivers were the PE characterization factor, the PE in the container, and the AgNP loading. The AgNP loading refers to the concentration of AgNP on the CWF based on the mass of AgNP applied per filter.^24^ The component with the highest influence on the environmental impact of the AgNP CWF system was the plastic bucket that holds the water that filters through the CWF. The AgNP coating had more influence on surface water due to the shorter AgNP lifespan, which results in more frequent AgNP recoating in response to the higher turbidity of the water.

For the UV mercury lamp system, the key drivers were UV mercury lamp lifespan, UV mercury lamp impact factor, aluminum impact factor, aluminum foil weight, and PE from storage. The key drivers of the UV mercury lamp system primarily revolved around capital requirements and lamp replacement. The UV lamps are key drivers of GWP and can be attributed to the lamp’s mercury content and the release of mercury into the environment during disposal.^29^ For the UV LED system, the key drivers were the LED characterization factor, PE characterization factor, stainless steel characterization factor, LED lifespan, stainless steel weight, UV LED weight, and PE weight. Both UV systems were impacted by the lifespan of the lamps and LEDs, as lamp replacement is necessary over time.

Overall, the results from the sensitivity analysis highlight the influence of assumptions on the financial and environmental sustainability of the POU technologies. The identification of key drivers can also guide technology developers in areas to focus on for research and improvement. For instance, when deploying POU chlorination using WaterGuard or similar products as a source of NaClO, the desired dose of NaClO will be an important factor to consider while adjusting for cost and environmental impacts. The cost of the AgNP CWF is primarily impacted by labor to manufacture the filters, suggesting that exploring mass production methods may further reduce the costs. Lowering the unit cost is a key area for improving the cost of both UV systems. The negative Spearman’s rank correlation of GWP impact of lamp and LED lifespan on GWP indicates that enhancing lifespan can increase environmental sustainability. These key drivers can provide a potential pathway for technology developers and manufacturers to improve the sustainability of POU technologies.

### 3.3 Short to Long-Term Adoption of POU Technologies

To assess the sustainability of the POU technologies over different adoption lifetimes, the study explored the impact of the length of adoption on the cost and environmental impacts. The adoption lifetime refers to the expected duration in years for which a household is likely to use a specific POU technology. In some cases, a POU technology may be deployed for short-term interventions, such as disaster relief efforts, or for long-term usage and treatment interventions, particularly in developing regions. For each POU technology, the study analyzed the cost and environmental impacts associated with adoption and usage periods ranging from 1 to 15 years. Across all POU technologies, a consistent trend was observed: as the adoption lifetime increased, the yearly per capita cost and environmental impact decreased. This trend is expected and more pronounced with technologies with high capital requirements since they are spread over the adoption lifetime in our analyses.^30,48^ This overall trend indicates that POU technologies exhibit greater sustainability with long-term adoption and usage. Long-term adoption is advantageous because it allows for the spreading out of costs and environmental impact over a greater number of years, as opposed to investing in a technology and using it only for a short period. However, it is important to note that the extent of cost and environmental impact reduction with longer lifetimes varies significantly among the different POU technologies.

**Figure 3:**
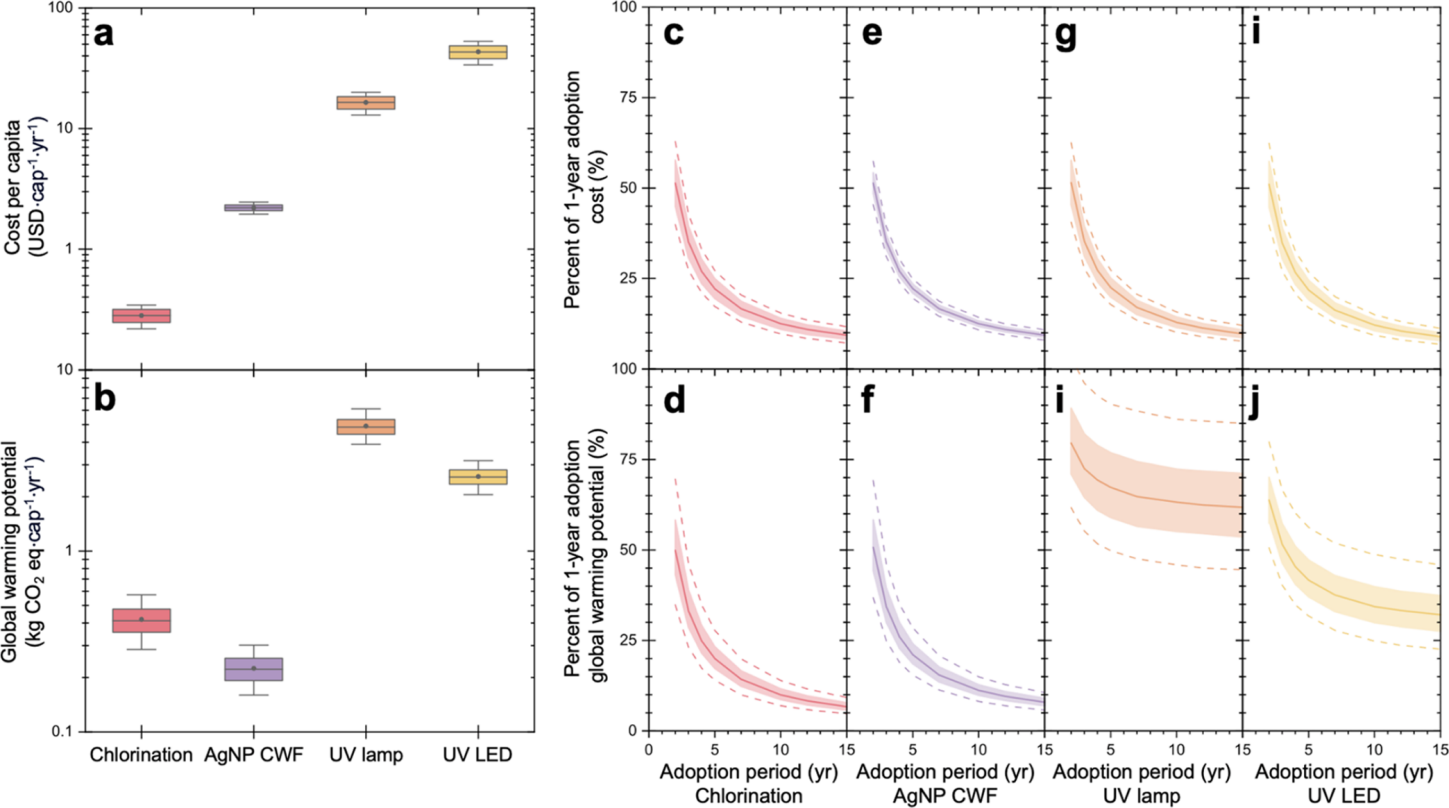
Costs (a) and global warming potential (b) for the technologies for a 1 year adoption period. Impact of short (2 year)- to long (15 year)-term adoption of POU technologies on cost (c, e, g, i) and global warming potential is shown for chlorination (c, d), AgNP CWF (e, f), UV lamp (g, i), and UV LED (i, j). These indicators are normalized to their respective median from the 1 year adoption period to show their relative yearly cost and global warming potential as the adoption period increases. Costs and impacts are annualized over the lifetime of the systems. The box and whisker plots show the median values (center line), 25th and 75th percentiles (bottom and top of box), 5th and 95th percentiles (lower and upper whiskers), and means (point). For the adoption period results, the median values are plotted as the center line, 25th and 75th percentiles are plotted in the shaded regions, and fifth and 95th percentiles are plotted with the dashed lines from the uncertainty analysis of 10,000 Monte Carlo simulations. Note that the household size was set to 4 people to focus on how adoption period can influence cost and environmental impacts.

The net costs and GWP for all four technologies during a 1 year adoption period (Figure 3a,b) were used to normalize results to their respective median from different adoption periods (Figure 3c–j). For POU chlorination, all values were lower with longer-term adoption. In the case of short-term adoption (1 year), the POU chlorination system had a median net cost of 0.42 USD·cap^–1^·yr^–1^ and median net GWP of 0.62 kg of CO_2_ eq·cap^–1^·yr^–1^. However, for long-term adoption (15 years) the net cost decreased to 9.31% of the 1 year adoption cost. On the other hand, the GWP for 15 years decreased to 6.67% of the 1 year adoption scenario. These reductions in cost and GWP with longer adoption periods are due to the distribution of capital requirements associated with the 20 L jerry can over the extended lifetime of the system. It does appear that both indicators level out at higher adoption periods, which can be attributed to the continuous need for consumables (i.e., NaClO) to run the system.

The AgNP CWF system exhibited the lowest GWP and the second lowest costs compared with all other technologies across the entire range of adoption lengths (Figure 3). Specifically, for a 1 year adoption term, the estimated net cost was 3.31 USD·cap^–1^·yr^–1^ and the net GWP was 0.33 kg of CO_2_ eq·cap^–1^·yr^–1^. Both the net cost and GWP significantly decreased as the adoption period increased from 1 to 5 years. At a 15 year adoption term, the estimated net cost and GWP were 9.31 and 7.92% of the 1 year adoption, respectively (Figure 3e,f). Therefore, for both short-term and long-term adoption, the AgNP CWF system appears to be a viable option. This system has the potential to be the most sustainable choice, considering both cost and environmental impacts.

Similarly, both UV systems had a pronounced decline in cost and a moderate decline in GWP with an increase in adoption lifetime. This finding can be attributed to the higher capital cost requirements associated with these advanced systems. In the case of the UV mercury lamp system, a 1 year adoption period was associated with a net cost of 24.59 USD·cap^–1^·yr^–1^ and a GWP of 7.28 kg CO_2_ eq·cap^–1^·yr^–1^ (Figure 3). However, the cost decreased significantly after approximately 5 years, with a 15 year adoption period resulting in a net cost of 9.74% of the 1 year adoption cost (Figure 3g). On the other hand, the 15 year adoption period resulted in 61.81% of the 1 year adoption GWP (Figure 3i). This only moderate reduction can be attributed to the GWP from the mercury lamps that require replacement throughout the adoption period.

The UV LED system had the highest cost of all of the POU technologies over the entire range of adoption periods (Figure 3a). A 1 year adoption was associated with a net cost of 64.92 USD·cap^–1^·yr^–1^, while a 15 year adoption yielded a net cost that was 8.95% of the 1 year adoption cost (Figure 3i). The GWP for a 1 year adoption was 4.15 kg of CO_2_ eq·cap^–1^·yr^–1^ and 32.10% of the 1 year adoption GWP (Figure 3j). The moderate reduction in GWP with adoption period for the UV LED system can also be attributed to the required lamp replacement. Overall, the drastic reduction in costs versus moderate reduction in GWP with adoption period for the UV-based system presents trade-offs in their adoption. It is notable that the local supply chain should be considered for sustained adoption as affordability for maintenance and continuous purchase of consumables and spare parts for technology can impact long-term usage.^30,48^ These results suggest that long-term adoption is the preferred approach when the costs of UV systems.

### 3.4 Implications on Technology Deployments

**Figure 4:**
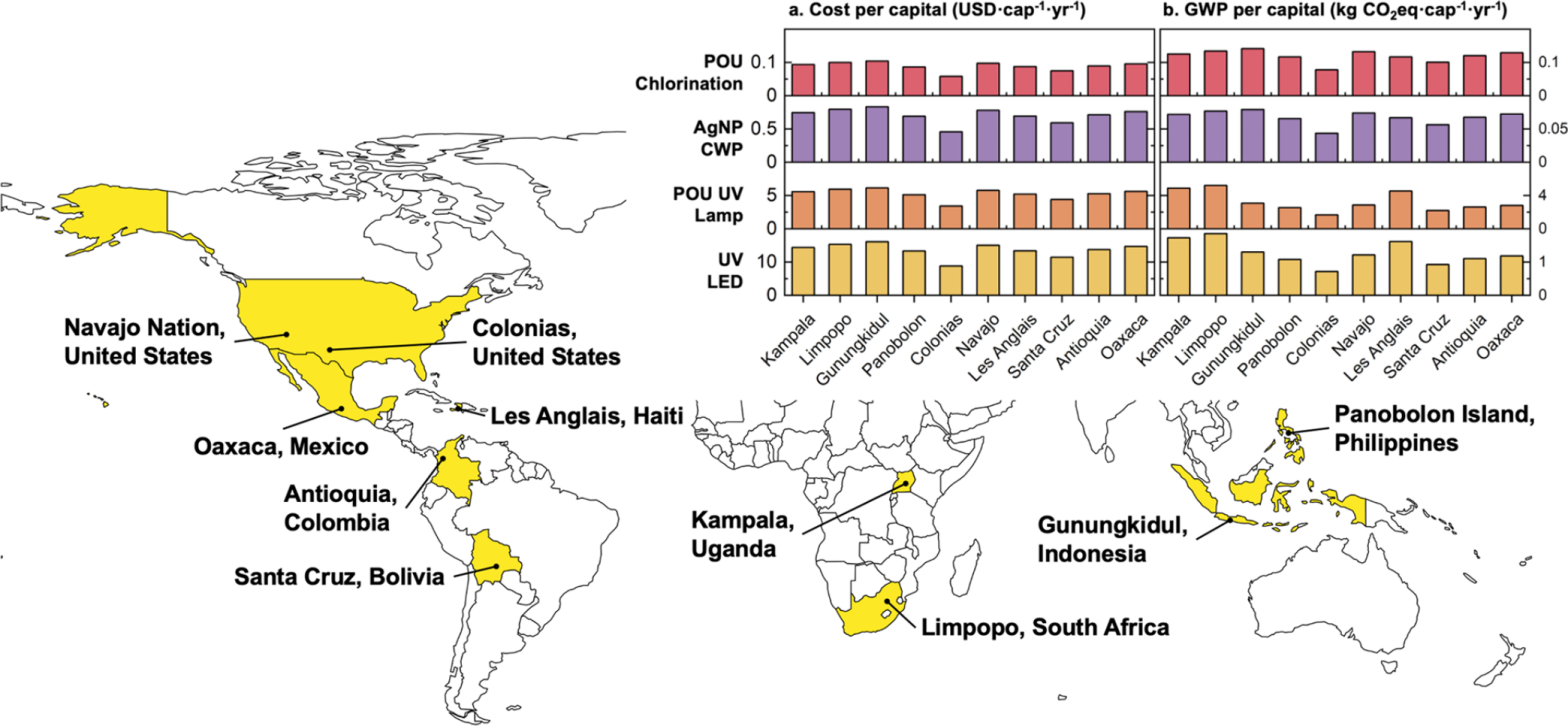
Location-specific costs (a) and global warming potential (GWP) (b) of the four POU technologies in 10 communities (highlighted in yellow) across the world.

As communities across the world are characterized by their unique economic, environmental, and social situations, location-specific parameters beyond technology specifications may also have substantial impacts on the overall sustainability. To explore these potential implications, 10 communities from four continents were included in a contextual analysis where TEA and LCA of the four POU technologies were performed with community and/or region-level demographic, water quality, and energy data (Table S7). Consistent with previous results, POU chlorination and AgNP CWF had much lower costs and GWP than UV lamp and UV LED, regardless of the deployment site (Figure 4). However, different trends were observed depending on the specific type of technology. For POU chlorination and AgNP CWF where the capital cost and construction of the equipment were cost and environmental impact drivers, per capita cost and GWP were found to be negatively correlated to the size of the household, with Colonias in the United States (household size of 6.48) and Santa Cruz, Bolivia (household size of 5) on the lower end and Gunungkidul, Indonesia, Limpopo, South Africa, Navajo Nation, United States, and Oaxaca, Mexico on the higher end (household size <4). For the two UV technologies, however, different trends were found for cost vs GWP, which were also correlated to both the water quality and the electricity profile of the community. For example, for Gunungkidul, Indonesia, which had the highest costs and GWP for POU chlorination and AgNP CWF (smallest household), though it still had the highest cost for UV lamp and UV LED, the GWP of these two UV systems were lower than those of Limpopo, South Africa (highest among all), Kampala, Uganda, and Les Anglais, Haiti. This change in trend was due to Gunungkidul’s comparably lower turbidity (0.36 NTU,^39^ would allow longer equipment lifetime and less electricity consumption) and a cleaner (0.687 kg CO_2_eq·kWh^–1^,^49^ vs 1.014 kg CO_2_eq·kWh^–1^ for Limpopo, South Africa^50^) grid in Indonesia. Notably, electricity was not identified as a driver for GWP in the sensitivity analysis, likely due to the narrower ranges considered previously (0.52 kg of CO_2_ eq·kWh^–1^ to 0.87). Finally, it should be noted that as these communities have very limited income, cost is nonetheless still likely to be the largest hurdle for the adoption of the UV technologies, which were found to be orders of magnitude higher than POU chlorination and AgNP CWF.

## 4 Conclusions

The QSD framework was leveraged in this study to compare the performance of POU technologies in terms of cost and environmental impacts. Based on the economic analysis, the POU chlorination system had the lowest net cost, while the UV LED system had the highest net cost, considering the baseline general assumptions. In terms of environmental impacts, the AgNP CWF system exhibited the lowest GWP, whereas the UV mercury lamp system had the highest environmental impacts, again based on the baseline general assumptions. If the motivation for selecting a technology is affordability, especially in low-income areas, POU chlorination would be appropriate for short-term adoption, while AgNP CWF may be more suitable for long-term adoption with moderately higher cost and lower dependence on the supply chain. On the other hand, if GWP is the deciding factor for selecting a technology, AgNP CWF would be appropriate based on the reported low environmental impacts, as revealed in this study. It is worth noting that the AgNP CWF is user-friendly; however, the process of recoating the AgNPs onto the CWF will require expert assistance, compared to POU chlorination, which households can easily use without needing expert involvement. On the UV systems, our findings indicate that UV LED had the higher cost under all adoption periods, but its GWP was lower compared to UV mercury lamps due to the disposal phase of the mercury of the lamps.^29^ However, due to the electricity demand, both UV systems would be less effective in regions where the electricity supply is not adequate or unavailable.

With regard to water quality, owing to the increased requirement for replaceable components or consumables to achieve effective disinfection, more turbid water would lead to a higher net GWP for all POU technologies. When the sustainability of technologies is evaluated, developers should evaluate the impact of different water sources. Further, the change in water quality would also propagate effects on sustainability drivers, such as the case where the NaClO dosage needs to be adjusted to align with the turbidity of the raw water. When a community has multiple potential water sources, it is important to understand the trade-offs in treatment costs and environmental impacts. Moreover, this study also revealed the significance of considering location-specific parameters for technology deployment. Using data specific to 10 communities across the world, we showed the variations in the cost and GWP of these four POU technologies.

Meanwhile, it is important to acknowledge that the results and findings in this study are under a set of assumptions derived from manufacturer recommendations, published reports, and scientific papers. The social acceptance and willingness to consistently use these POU technologies would have a significant impact on the realized sustainability of POU water treatment. The specific results and outcomes can vary depending on the changes in key assumptions and parameters that drive sustainability. Other water quality metrics could be considered in future work. Moreover, the inclusion of additional decision variables, contextual parameters, and technological parameters may yield different outcomes. For instance, factors such as the cost of water transportation from source or the energy required for groundwater pumping may have an impact on the magnitude of the results. Incorporating these additional parameters or modifying existing ones in future analyses can yield more context-specific and informed results. Thus, this study can serve as a foundation for future researchers and entities interested in understanding the relative sustainability of different POU technologies. Finally, while this study focused on four selected POU technologies, the framework employed can be extended to explore other POU technologies, including novel and emerging ones, that need to be evaluated prior to deploying. Therefore, this study has the potential to help inform research, development, and deployment of POU disinfection technologies considering decision variables, technological parameters, and contextual parameters.

## Supporting Information Available

Data set for the POU chlorination script; data set for the AgNP CWF script; data for the UV mercury lamp system script; data for the UV LED system script; data for the raw water scripts for both water types; data for global warming potential (GWP) assumptions; data for contextual analysis; quantitative sustainable design (QSD) methodology; visual representation of POU chlorination; visual representation of silver nanoparticle-enabled ceramic water filters; visual representation of UV disinfection with mercury lamps; visual representation of UV disinfection with LEDs; system boundaries and foreground inventory for the four point-of-use disinfection systems; and Spearman’s rank correlation for net cost and GWP for all POU technologies with groundwater (PDF)

## References

[ref1] Nations, U.. Water and Sanitation United Nations Sustainable Developmenthttps. //www.un.org/sustainabledevelopment/water-and-sanitation/ (accessed February 28, 2024).

[ref2] CDC. Disease Threats and Global WASH Killers Centers for Disease Control and Preventionhttps 2020//www.cdc.gov/healthywater/global/WASH.html (accessed February 28, 2024).

[ref3] Garrett V.; Ogutu P.; Mabonga P.; Ombeki S.; Mwaki A.; Aluoch G.; Phelan M.; Quick R. E. Diarrhoea Prevention in a High-Risk Rural Kenyan Population through Point-of-Use Chlorination, Safe Water Storage, Sanitation, and Rainwater Harvesting. Epidemiol. Infect. 2008, 136 (11), 1463–1471. 10.1017/S095026880700026X. 18205977 PMC2870746

[ref4] Torbaghan M. E.; Burrow M. Small Town Water Supply Infrastructure Costs; Institute of Development Studies, 2019.

[ref5] Fonseca C. The Death of the Communal Handpump? Rural Water and Sanitation Household Costs in Lower-Income Countries, Ph.D. Thesis; Cranfield University, School of Applied Sciences, Water Sciences: Cranfield, UK, 2014. http://dspace.lib.cranfield.ac.uk/handle/1826/8512.

[ref6] Nyarko K. B.; Dwumfour-Asare B.; Appiah-Effah E.; Moriarty P. B. Costs of Delivering Water Service in Rural Areas and Small Towns in Ghana: Paper Presented at the IRC Symposium ‘ Pumps, Pipes and Promises: Costs, Finances and Accountability for Sustainable WASH Services’ in The Hague, The Netherlands from 16 - 18 Nove,. 2010.

[ref7] Pooi C. K.; Ng H. Y. Review of Low-Cost Point-of-Use Water Treatment Systems for Developing Communities. npj Clean Water 2018, 1 (1), 11 10.1038/s41545-018-0011-0.

[ref8] Bailey E. S.; Beetsch N.; Wait D. A.; Oza H. H.; Ronnie N.; Sobsey M. D. Methods, Protocols, Guidance and Standards for Performance Evaluation for Point-of-Use Water Treatment Technologies: History, Current Status, Future Needs and Directions. Water 2021, 13 (8), 1094 10.3390/w13081094.

[ref9] Graf J.; Serge Z. T.; Kemka N.; Niyitegeka D.; Meierhofer R.; Gangoué-Piéboji J. Health Gains from Solar Water Disinfection (SODIS): Evaluation of a Water Quality Intervention in Yaoundé, Cameroon. J. Water Health 2010, 8, 779–796. 10.2166/wh.2010.003. 20705988

[ref10] Mikelonis A. M.; Rowles L. S.; Lawler D. F. The Effects of Water Chemistry on the Detachment and Dissolution of Differently Stabilized Silver Nanoparticles from Ceramic Membranes. Environ. Sci.: Water Res. Technol. 2020, 6 (5), 1347–1356. 10.1039/C9EW01141B.

[ref11] Rowles L. S.; Tso D.; Dolocan A.; Kirisits M. J.; Lawler D. F.; Saleh N. B. Integrating Navajo Pottery Techniques To Improve Silver Nanoparticle-Enabled Ceramic Water Filters for Disinfection. Environ. Sci. Technol. 2023, 57 (44), 17132–17143. 10.1021/acs.est.3c03462. 37870911

[ref12] Clasen T.; Brown J.; Suntura O.; Collin S. Safe Household Water Treatment and Storage Using Ceramic Drip Filters: A Randomised Controlled Trial in Bolivia. Water Sci. Technol. 2004, 50 (1), 111–115. 10.2166/wst.2004.0033. 15318495

[ref13] Mellor J.; Abebe L.; Ehdaie B.; Dillingham R.; Smith J. Modeling the Sustainability of a Ceramic Water Filter Intervention. Water Res. 2014, 49, 286–299. 10.1016/j.watres.2013.11.035. 24355289 PMC3924855

[ref14] Rogers E.; Tappis H.; Doocy S.; Martínez K.; Villeminot N.; Suk A.; Kumar D.; Pietzsch S.; Puett C. Costs and Cost-Effectiveness of Three Point-of-Use Water Treatment Technologies Added to Community-Based Treatment of Severe Acute Malnutrition in Sindh Province, Pakistan. Global Health Action 2019, 12 (1), 1568827 10.1080/16549716.2019.1568827. 30888265 PMC6427553

[ref15] Busse M. M.; Hawes J. K.; Blatchley E. R. I. Comparative Life Cycle Assessment of Water Disinfection Processes Applicable in Low-Income Settings. Environ. Sci. Technol. 2022, 56 (22), 16336–16346. 10.1021/acs.est.2c02393. 36215720

[ref16] Walsh T.; Mellor J. Comparative Life Cycle Assessment of Four Commonly Used Point-of-Use Water Treatment Technologies. J. Water, Sanit. Hyg. Dev. 2020, 10 (4), 862–873. 10.2166/washdev.2020.158.

[ref17] Li Y.; Trimmer J. T.; Hand S.; Zhang X.; Chambers K. G.; Lohman H. A. C.; Shi R.; Byrne D. M.; Cook S. M.; Guest J. S. Quantitative Sustainable Design (QSD) for the Prioritization of Research, Development, and Deployment of Technologies: A Tutorial and Review. Environ. Sci.: Water Res. Technol. 2022, 8 (11), 2439–2465. 10.1039/D2EW00431C.

[ref18] Quantitative Sustainable Design (QSD) Group. EXPOsan: EXPOsition of sanitation and resource recovery systems. https://github.com/QSD-Group/EXPOsan (accessed February 28, 2024).

[ref19] QSDsan: Quantitative Sustainable Design for Sanitation and Resource Recovery Systems 2022 https://github.com/QSD-Group/QSDsan (accessed February 28, 2024).

[ref20] Li Y.; Zhang X.; Morgan V. L.; Lohman H. A. C.; Rowles L. S.; Mittal S.; Kogler A.; Cusick R. D.; Tarpeh W. A.; Guest J. S. QSDsan: An Integrated Platform for Quantitative Sustainable Design of Sanitation and Resource Recovery Systems. Environ. Sci.: Water Res. Technol. 2022, 8 (10), 2289–2303. 10.1039/D2EW00455K.

[ref21] EXPOsan/exposan/pou_disinfection at main · QSD-Group/EXPOsanGitHub. https://github.com/QSD-Group/EXPOsan/tree/main/exposan/pou_disinfection (accessed February 03, 2024).

[ref22] Quantitative Sustainable Design (QSD) Group GitHub. https://github.com/QSD-Group (accessed February 28, 2024).

[ref23] Nations U. Household Size and Composition around the World \textbar Population Division. https://www.un.org/development/desa/pd/content/household-size-and-composition-around-world (accessed 2024–02–28).

[ref24] UNICEF. Access to Drinking Water UNICEF DATA. https://data.unicef.org/topic/water-and-sanitation/drinking-water/ (accessed Febeuary 28, 2024).

[ref25] Mohamed H.; Brown J.; Njee R. M.; Clasen T.; Malebo H. M.; Mbuligwe S. Point-of-Use Chlorination of Turbid Water: Results from a Field Study in Tanzania. J. Water Health 2015, 13 (2), 544–552. 10.2166/wh.2014.001. 26042985

[ref26] Mwabi J. K.; Mamba B. B.; Momba M. N. B. Removal of *Escherichia Coli* and Faecal Coliforms from Surface Water and Groundwater by Household Water Treatment Devices/Systems: A Sustainable Solution for Improving Water Quality in Rural Communities of the Southern African Development Community Region. Int. J. Environ. Res. Public Health 2012, 9 (1), 139–170. 10.3390/ijerph9010139. 22470284 PMC3315086

[ref27] Ren D.; Colosi L. M.; Smith J. A. Evaluating the Sustainability of Ceramic Filters for Point-of-Use Drinking Water Treatment. Environ. Sci. Technol. 2013, 47 (19), 11206–11213. 10.1021/es4026084. 23991752

[ref28] Lui G. Y.; Roser D.; Corkish R.; Ashbolt N. J.; Stuetz R. Point-of-Use Water Disinfection Using Ultraviolet and Visible Light-Emitting Diodes. Sci. Total Environ. 2016, 553, 626–635. 10.1016/j.scitotenv.2016.02.039. 26967007

[ref29] Chatterley C.; Linden K. Demonstration and Evaluation of Germicidal UV-LEDs for Point-of-Use Water Disinfection. J. Water Health 2010, 8 (3), 479–486. 10.2166/wh.2010.124. 20375477

[ref30] Sobsey M. D.; Stauber C. E.; Casanova L. M.; Brown J. M.; Elliott M. A. Point of Use Household Drinking Water Filtration: A Practical, Effective Solution for Providing Sustained Access to Safe Drinking Water in the Developing World. Environ. Sci. Technol. 2008, 42 (12), 4261–4267. 10.1021/es702746n. 18605542

[ref31] Stumm W.; Morgan J. J. Aquatic Chemistry: Chemical Equilibria and Rates in Natural Waters; Wiley, 1996.

[ref32] Jennergren P. A Tutorial on the Discounted Cash Flow Model for Valuation of CompaniesStockholm School of Economics 2011 https://econpapers.repec.org/paper/hhbhastba/0001.htm.

[ref33] Bare J. TRACI 2.0: The Tool for the Reduction and Assessment of Chemical and Other Environmental Impacts 2.0. Clean Technol. Environ. Policy 2011, 13 (5), 687–696. 10.1007/s10098-010-0338-9.

[ref34] Ecoinvent Online Access - Ecoinvent Ecoinvent Database 2020 https://ecoinvent.org/the-ecoinvent-database/online-access/ (accessed February 28, 2024).

[ref35] McKay M. D.; Beckman R. J.; Conover W. J. A Comparison of Three Methods for Selecting Values of Input Variables in the Analysis of Output from a Computer Code. Technometrics 1979, 21 (2), 239–245. 10.2307/1268522.

[ref36] Trimmer J. T.; Lohman H. A. C.; Byrne D. M.; Houser S. A.; Jjuuko F.; Katende D.; Banadda N.; Zerai A.; Miller D. C.; Guest J. S. Navigating Multidimensional Social–Ecological System Trade-Offs across Sanitation Alternatives in an Urban Informal Settlement. Environ. Sci. Technol. 2020, 54 (19), 12641–12653. 10.1021/acs.est.0c03296. 32822180

[ref37] Bwire G.; Sack D. A.; Kagirita A.; Obala T.; Debes A. K.; Ram M.; Komakech H.; George C. M.; Orach C. G. The Quality of Drinking and Domestic Water from the Surface Water Sources (Lakes, Rivers, Irrigation Canals and Ponds) and Springs in Cholera Prone Communities of Uganda: An Analysis of Vital Physicochemical Parameters. BMC Public Health 2020, 20 (1), 1128 10.1186/s12889-020-09186-3. 32680495 PMC7368733

[ref38] Edokpayi J. N.; Rogawski E. T.; Kahler D. M.; Hill C. L.; Reynolds C.; Nyathi E.; Smith J. A.; Odiyo J. O.; Samie A.; Bessong P.; Dillingham R. Challenges to Sustainable Safe Drinking Water: A Case Study of Water Quality and Use across Seasons in Rural Communities in Limpopo Province, South Africa. Water 2018, 10 (2), 159 10.3390/w10020159. 30595910 PMC6310213

[ref39] Matthies K.; Schott C.; Anggraini A. K.; Silva A.; Diedel R.; Mühlebach H.; Fuchs S.; Obst U.; Brenner-Weiss G. Drinking Water Treatment for a Rural Karst Region in Indonesia. Appl. Water Sci. 2016, 6 (3), 309–318. 10.1007/s13201-016-0423-2.

[ref40] Espaldon A. E.; Yu Jeco-Espaldon B. M.; Sado T.; Tenebro C. P.; Dalisay D. S.; Oguma K.; Saludes J. P. Groundwater Quality Analyses in Off-Grid Tropical Island. Water Environ. J. 2022, 36 (4), 644–655. 10.1111/wej.12804.

[ref41] Rowles L. S.; Whittaker T.; Ward P. M.; Araiza I.; Kirisits M. J.; Lawler D. F.; Saleh N. B. A Structural Equation Model to Decipher Relationships among Water, Sanitation, and Health in Colonias-Type Unincorporated Communities. Environ. Sci. Technol. 2020, 54 (24), 16017–16027. 10.1021/acs.est.0c05355. 33259189

[ref42] Rowles III L. S.; Hossain A. I.; Ramirez I.; Durst N. J.; Ward P. M.; Kirisits M. J.; Araiza I.; Lawler D. F.; Saleh N. B. Seasonal Contamination of Well-Water in Flood-Prone Colonias and Other Unincorporated U.S. Communities. Sci. Total Environ. 2020, 740, 140111 10.1016/j.scitotenv.2020.140111. 32562995

[ref43] Roy M. A.; Arnaud J. M.; Jasmin P. M.; Hamner S.; Hasan N. A.; Colwell R. R.; Ford T. E. A Metagenomic Approach to Evaluating Surface Water Quality in Haiti. Int. J. Environ. Res. Public Health 2018, 15 (10), 2211 10.3390/ijerph15102211. 30309013 PMC6209974

[ref44] Rowles L. S.; Alcalde R.; Bogolasky F.; Kum S.; Diaz-Arriaga F. A.; Ayres C.; Mikelonis A. M.; Toledo-Flores L. J.; Alonso-Gutiérrez M. G.; Pérez-Flores M. E.; Lawler D. F.; Ward P. M.; Lopez-Cruz J. Y.; Saleh N. B. Perceived versus Actual Water Quality: Community Studies in Rural Oaxaca, Mexico. Sci. Total Environ. 2018, 622–623, 626–634. 10.1016/j.scitotenv.2017.11.309. 29223086

[ref45] Peláez M. A. Z. Implementing a UV Disinfection System in a Low-Income Area of Bolivia; University of Alberta: South America, 2011.

[ref46] Botero L.; Galeano L.; Montoya L. J.; Machado A.; Byrne J. A.; Fernandez-Ibañez P.; Hincapié M. *Aeromonas Hydrophila* in Surface Water and Their Removal Using a POU Technology for Drinking in Rural Communities. Environ. Adv. 2023, 13, 100425 10.1016/j.envadv.2023.100425.

[ref47] Water Guard. Engineering For Change. https://www.engineeringforchange.org/solutions/product/waterguard/ (accessed February 28, 2024).

[ref48] Wood S.; Foster J.; Kols A. Understanding Why Women Adopt and Sustain Home Water Treatment: Insights from the Malawi Antenatal Care Program. Soc. Sci. Med. 2012, 75 (4), 634–642. 10.1016/j.socscimed.2011.09.018. 22051403

[ref49] International Renewable Energy Agency. Energy Profile - Indonesia. https://www.irena.org/-/media/Files/IRENA/Agency/Statistics/Statistical_Profiles/Asia/Indonesia_Asia_RE_SP.pdf (accessed February 28, 2024).

[ref50] International Renewable Energy Agency. Energy Profile - South Africa. https://www.irena.org/-/media/Files/IRENA/Agency/Statistics/Statistical_Profiles/Africa/South-Africa_Africa_RE_SP.pdf (accessed February 28, 2024).

