# Supplementary material for: Assessing the Relative Sustainability of Point-of-Use Water Disinfection Technologies for Off-Grid Communities
Source: ACS Environ Au. 2024 Jul 9;4(5):248–59. doi: 10.1021/acsenvironau.4c00017 (PMC11413886; doi:10.1021/acsenvironau.4c00017)
Supplement: Supplementary file 1 — vg4c00017_si_001.pdf [file vg4c00017_si_001.pdf]

Supporting Information for

Assessing the relative sustainability of point-of-use water disinfection technologies for off-grid communities

Bright C. Elijah[1]; Ali Ahmad[2]; Yalin Li [2]; Jaime Plazas-Tuttle[3]; Lewis S. Rowles[1]*

[1]*Department of Civil Engineering and Construction, Georgia Southern University, Statesboro, Georgia, 30458, United States*

[2]*Department of Civil and Environmental Engineering, Rutgers, The State University of New Jersey, Piscataway, New Jersey, 08854, United States*

[3]*Department of Civil and Environmental Engineering, Universidad de los Andes, Bogotá, 111711, Colombia*

**Corresponding author: Lewis S. Rowles, email: lrowles@georgiasouthern.edu, phone: (912) 478-0772*

Table of Contents (17 total pages):

Table S1. Data set for the POU chlorination script
Table S2. Dataset for the AgNP CWF script
Table S3. Data for the UV mercury lamp system script
Table S4. Data for the UV LED system script
Table S5. Data for the raw water scripts for both water types
Table S6. Data for global warming potential (GWP) assumptions
Table S7. Data for contextual analysis
Figure S1. Quantitative sustainable design (QSD) methodology
Figure S2. Visual representation of POU chlorination
Figure S3. Visual representation of silver nanoparticle-enabled ceramic water filters
Figure S4. Visual representation of UV disinfection with mercury lamps
Figure S5. Visual representation of UV disinfection with LEDs
Figure S6. System boundaries and foreground inventory for the four point-of-use disinfection systems
Figure S7. Spearman's rank correlation for net cost and GWP for all POU technologies with groundwater
Supporting References

Table S1. Data set for the POU chlorination script. The dataset covers materials cost and design parameters. The uncertainty added to the expected values is shown as low and high values with the distribution as noted.

parameter	unit	expected	low	high	distribution	references
**NaOCl _dose**	mg NaOCl/L	1.87	1.4025	2.3375	uniform	1
**contact_time**	min	30	22.5	37.5	uniform	1
**container_cost**	USD	1.6	1.2	2	uniform	2
**container_volume**	L	20			constant	1
**container_vol**	L/container	20			constant	1
**NaOCl _cost**	USD/kg	1.96	1.47	2.45	uniform	3
**NaOCl _volume**	L/bottle	0.15			constant	1
**NaOCl _density**	kg/L	1.21			constant	4
**rate_coefficient**		3.2185			constant	5
**intercept**		0.0845			constant	5
**PE_in_container**	kg	0.9	0.675	1.125	uniform	6
**PE_to_ NaOCl**	kgPE/kgNaOCl	0.096			constant	
**NaClO _bottle_mass**	kg	0.023	0.0172	0.0287	uniform	7
**steel_pole_mass**	kg	1			constant	
**operator_refill_cost**	USD/refill	10	9	11	uniform	

Table S2. Dataset for the AgNP CWF script. The dataset covers materials cost and design parameters. The uncertainty added to the expected values is shown as low and high values with the distribution as noted.

parameter	unit	expected	low	high	distribution	references
**CWF_cost**	USD	16.68	8	35	triangular	[8]
**Clay_mass**	kg	4.7	3.525	5.875	uniform	[8]
**Sawdust_mass**	kg	1.3	0.975	1.625	uniform	[8]
**CWF_water**	kg	2	1.5	2.5	uniform	[8]
**AgNP**	kg/filter	0.000064	9.6E-06	0.000096	uniform	[8]
**Electricity**	MJ	0.0324	0.0243	0.0405	uniform	[8]
**Brush_cost**	USD	0.4	0.3	0.5	uniform	[9]
**Wood_mass**	kg	6.1	4	9.4	triangular	[8]
**propane**	kg	0.9	0.55	1.3	triangular	[8]
**PE_in_container**	kg	0.9	0.675	1.125	uniform	[6]
**AgNP_low_lifetime**	yr	1	0.5	1.5	uniform	[10]
**AgNP_lifetime**	yr	3	1.5	4.5	uniform	[10]
**CWF_clay_cost**	USD/filter	0.99	0.7425	1.2375	uniform	[11]
**CWF_grog_cost**	USD/filter	0.18	0.135	0.225	uniform	[11]
**CWF_sawdust_cost**	USD/filter	0.9	0.675	1.125	uniform	[11]
**CWF_AgNP_cost_old**	USD/filter	10	7.5	12.5	uniform	[11]
**CWF_water_cost**	USD/filter	0.02	0.015	0.025	uniform	[11]
**CWF_wood_cost**	USD/filter	0.5	0.375	0.625	uniform	[11]
**CWF_labor_cost**	USD/filter	5.53	4.1475	6.9125	uniform	[11]
**CWF_additional_cost**	USD/filter	4	3	5	uniform	[11]
**CWF_AgNP_cost**	USD/kg	1250	1000	1500	triangular	[11]
**Argenol_AgNP_content**	fraction Ag	0.725	0.7	0.75	triangular	[12]
**CWF_bucket**	USD	2	1.5	2.5	uniform	[11]
**CWF_lid**	USD	1	0.75	1.25	uniform	[11]
**CWF_spout**	USD	1	0.75	1.25	uniform	[11]

Table S3. Date for the UV mercury lamp system script. The dataset covers materials cost and design parameters. The uncertainty added to the expected values is shown as low and high values with the distribution as noted.

parameter	unit	expected	low	high	distribution	references
**uv_dose**	mJ/cm^2	187	140.25	233.75	uniform	[13]
**UVT**	%	95			constant	[13]
**uv_unit_cost**	USD	90	67.5	112.5	uniform	[13]
**uv_flow**	L/min	9.46	7.095	11.825	uniform	[13]
**residence_time**	s	8			constant	[13]
**quartz_tube_volume**	L	1.54			constant	[13]
**uv_lamp_cost**	USD	26	19.5	32.5	uniform	[13]
**uv_pump_cost**	USD	75	56.25	93.75	uniform	
**lamp_life_span**	hr	2000	1500	2500	uniform	[13]
**uv_PVC**	kg	0.3844	0.2883	0.4805	uniform	[13]
**uv_aluminum_foil**	kg	0.3916	0.2937	0.4895	uniform	[13]
**storage_PE**	kg/container	0.9	0.675	1.125	uniform	[6]
**uv_storage_cost**	USD	1.6	1.2	2	uniform	[2]
**uv_electric_cost**	USD/L	0.000015	1.13E-05	1.88E-05	uniform	[13]
**uv_electric_demand**	W	30	22.5	37.5	uniform	[13]
**number_of_uv_lamps**	number of lamps	2			constant	[13]
**uv_water_volume**	L	20			constant	
**unit_volume**	L	20			constant	
**uv_slope**		0.205			constant	
**uv_slope**		0.45			constant	

Table S4. Data for the UV LED system script. The dataset covers materials cost and design parameters. The uncertainty added to the expected values is shown as low and high values with the distribution as noted.

parameter	unit	expected	low	high	distribution	references
**UVT**	%	95			constant	[14]
**uv_led_unit_cost**	USD	245	183.75	306	uniform	[14]
**uv_led_electric_cost**	USD	0.2	0.15	0.25	uniform	[14]
**uv_led_flow**	L/min	0.19	0.1425	0.2375	uniform	[15]
**residence_time**	S	8			constant	
**uv_led_cost**	USD	26	19.5	32.5	triangular	[16]
**uv_led_lifespan**	hr	10000	7500	12500	uniform	[14]
**Uv_led_pump_cost**	USD	75	56.25	93.75	uniform	
**uv_led_per_unit**		30			constant	[15]
**uv_led_storage_cost**	USD	1.6	1.2	2	uniform	[2]
**uv_led_quartz**	kg	0.0812	0.0609	0.1015	uniform	[15]
**StainlessSteel**	kg	1.3285	0.9964	1.6607	uniform	[15]
**uv_led_dose**	mJ/cm^2	187	95	215	uniform	[15]
**uv_led_slope**		0.217			constant	
**uv_led_intercept**		0.2			constant	
**led_electricity_demand**	W	23	11	35	uniform	[17]
**uv_quartz**	Kg	0.2331	0.175	0.2914	uniform	[15]
**unit_volume**	L	20			constant	
**uv_led_weight**	kg	0.0105	0.0079	0.0131	uniform	[18]
**uv_led_storage_PE**	kg/ container	0.9	0.675	1.125	uniform	[6]

Table S5. Data for the raw water scripts for both water types. The dataset covers water quality parameters. The uncertainty added to the expected values is shown as low and high values with the distribution as noted.

Groundwater						
**parameter**	**unit**	**expected**	**low**	**high**	**distribution**	**references**
**E_coli**	CFU/mg	200000	150000	250000	uniform	[11]
**Turbidity**	NTU	5	1	10	uniform	[19]
**TOC**	mg/L	5	1	10	uniform	[20]
**Ca**	mg/L	30	20	40	uniform	[13]
**Mg**	mg/L	30	20	40	uniform	[13]
**UVT**	%	80	72	88	uniform	[13]
**Surface water**						
**parameter**	**unit**	**expected**	**low**	**high**	**distribution**	**references**
**E_coli**	CFU/mg	200000	150000	250000	uniform	[11]
**Turbidity**	NTU	20	10	30	uniform	[19]
**TOC**	mg/L	10	5	15	uniform	[20]
**Ca**	mg/L	10	1	20	uniform	[13]
**Mg**	mg/L	10	1	20	uniform	[13]
**UVT**	%	80	72	88	uniform	[13]

Table S6. Data for global warming potential (GWP) assumptions. The GWP impact dataset covers all materials accounted for in all the POU technologies and their GWP. Uncertainty is added to each GWP datapoint, and the data source is Ecoinvent version 3.

ID	unit	GWP	expected	low	high	distribution	references
StainlessSteel	kg	kg CO2-eq	5.0231	3.767325	6.278875	uniform	ecoinvent 3
PE	kg	kg CO2-eq	2.7933	2.094975	3.491625	uniform	ecoinvent 3
PVC	kg	kg CO2-eq	2.4204	1.8153	3.0255	uniform	ecoinvent 3
UVlamp	kg	kg CO2-eq	0.98118	0.735885	1.226475	uniform	ecoinvent 3
Aluminum	kg	kg CO2-eq	15.106	11.3295	18.8825	uniform	ecoinvent 3
CWFClay	kg	kg CO2-eq	0.010238	0.007679	0.012798	uniform	ecoinvent 3
SilverNP	kg	kg CO2-eq	496.58	372.435	620.725	uniform	ecoinvent 3
Sawdust	kg	kg CO2-eq	0.022008	0.016506	0.02751	uniform	ecoinvent 3
Quartz	kg	kg CO2-eq	0.035012	0.026259	0.043765	uniform	ecoinvent 3
LED	kg	kg CO2-eq	247.43	185.5725	309.175	uniform	ecoinvent 3
Electricity	kWh	kg CO2-eq	0.69712	0.52284	0.8714	uniform	ecoinvent 3
NaClO	kg	kg CO2-eq	2.6287	1.971525	3.285875	uniform	ecoinvent 3
PE_stream	kg	kg CO2-eq	2.7933	2.094975	3.491625	uniform	ecoinvent 3

Table S7. Data for the contextual analysis. Baseline values were used for parameters not included in this table. For values with +/-, the average value was used; for communities where multiple data were collected (e.g., dry vs. wet season) or a range was provided, the upper bound of the value was used for the result to be conservative (CF: characterization factor).

Community	House hold size	*E. coli* [CFU/100 mL]	Turbidity [NTU]	Hardness [mg/L $CaCO_3$]	Electricity cost [$/kWh]	Electricity GWP CF [kg CO_2-eq/kWh]
Kampala, Uganda	4±1.8[21]	0.5-4839.2[22]	122.09± 23.78[22]	negligible[22]	0.166[23]	0.204[24]
Limpopo, South Africa	3.75	10-2000[25]	6.17-44.7[25]	219-1234[25]	0.100[26]	1.014[27]
Gunungkidul, Indonesia	3.56[28]	840-4880 (wet) [29]<br>17-200 (dry)[29]	0.36[29]	258[29]	0.075[30]	0.687[31]
Panobolon Island, Philippines	4.3[32]	564±884 (wet)[33]<br>92±48 (dry)[33]	9±17 (wet)[33]<br>5±10 (dry)[33]	142±104 (wet)[33]<br>0.0±0.1 (dry)[33]	0.164[34]	0.676[35]
Colonias, United States	6.48[36]	10-200[37]	negligible[36]	150-250[37]	0.126[36]	0.350[38]
Navajo Nation, United States	3.81[39]	2-30[39]	0.3[39]	53.95[39]	0.184[39]	0.352[38]
Les Anglais, Haiti	4.3	1-100[40]	39	200	0.098[41]	0.709[42]
Santa Cruz, Bolivia	5[43]	93-4300[43]	5.6-14.8[43]	492[43]	0.163[44]	0.299[45]
Antioquia, Colombia[a]	4.17[46]	100±121[47]<br>29±36[47]	7.1±1.5[47]<br>6.5± 4.2[47]	assumed to be the same as surface water[b]	0.146[48]	0.148[49]
Oaxaca, Mexico	3.9[50]	0-1500[50]	0.5-2[50]	55-407[50]	0.120[51]	0.292[52]

[a] Two sites were discussed in the source.
[b] Default hardness value for surface water in this work (20 mg/L as $CaCO_3$) were used due to the unavailability of data in the community.

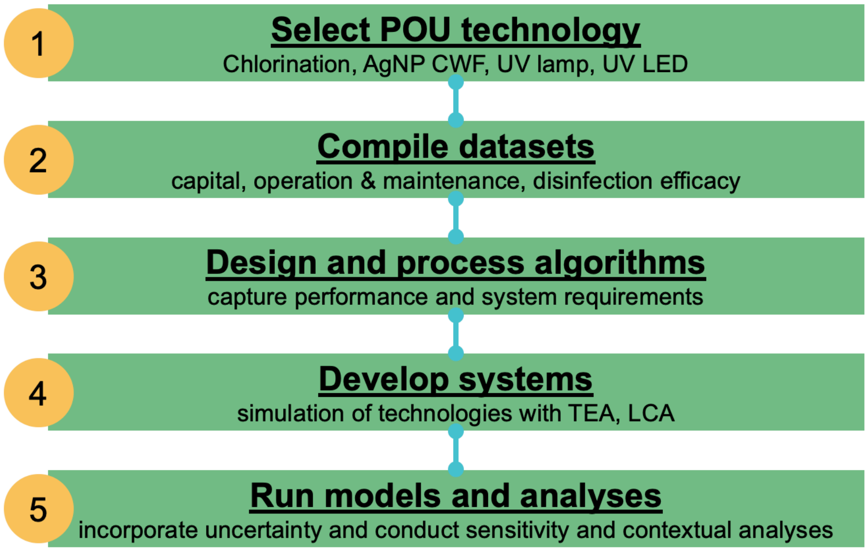

Figure S1. Quantitative sustainable design (QSD) methodology leveraged to develop the models for this work by selecting POU technologies, compiling datasets, developing design and process algorithms, developing systems, and running models and analyses.

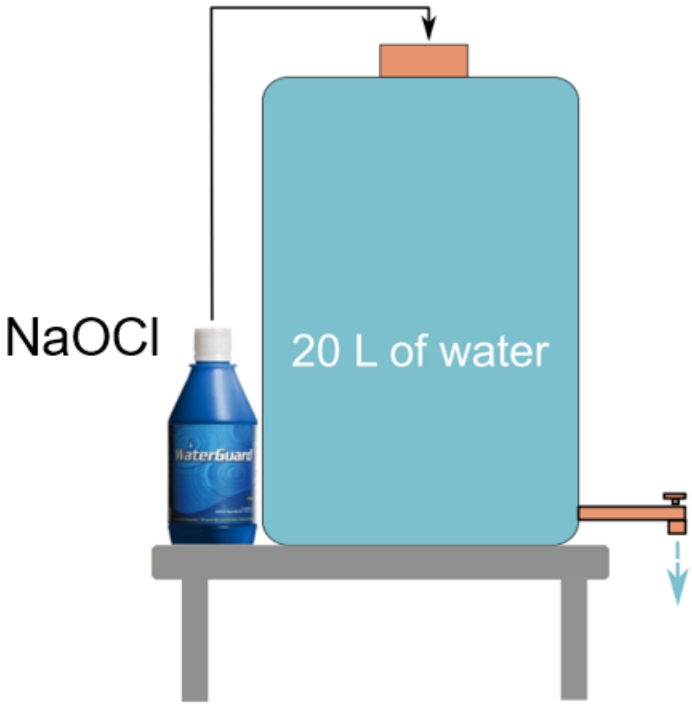

Figure S2. Visual representation of POU chlorination using NaOCl from WaterGuard. This set-up is designed to be easy to use as the cap of the WaterGuard bottle is used to dose the NaOCl solution to 20 L of water for disinfection.

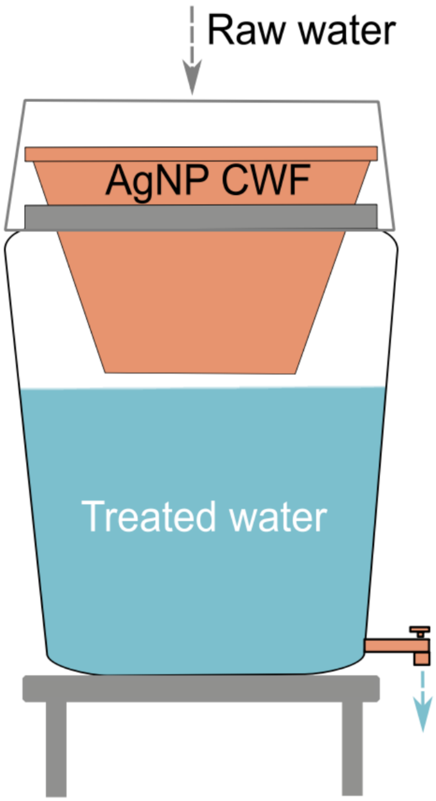

Figure S3. Visual representation of silver nanoparticle-enabled ceramic water filters (AgNP CWF).[11] These point-of-use (POU) disinfection technology have dual mechanisms to remove microorganisms through filtration and chemical disinfection. They are commonly produced with local clays near target communities.

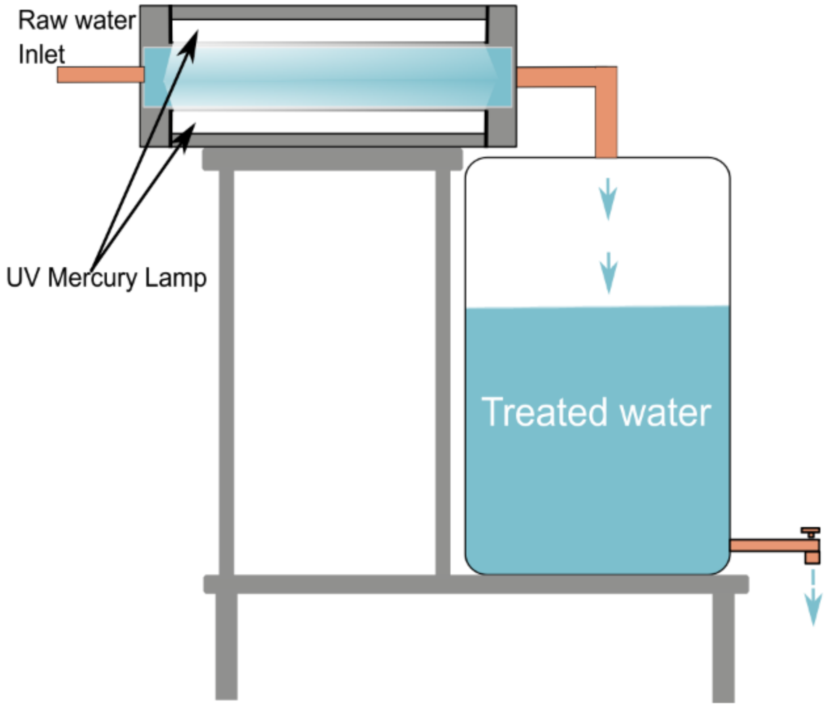

Figure S4. Visual representation of UV disinfection with mercury lamps. This set-up has water that flows through the UV system with two mercury lamps as the source of UV transmission for disinfection. Treated water is then stored in a container for use as needed.

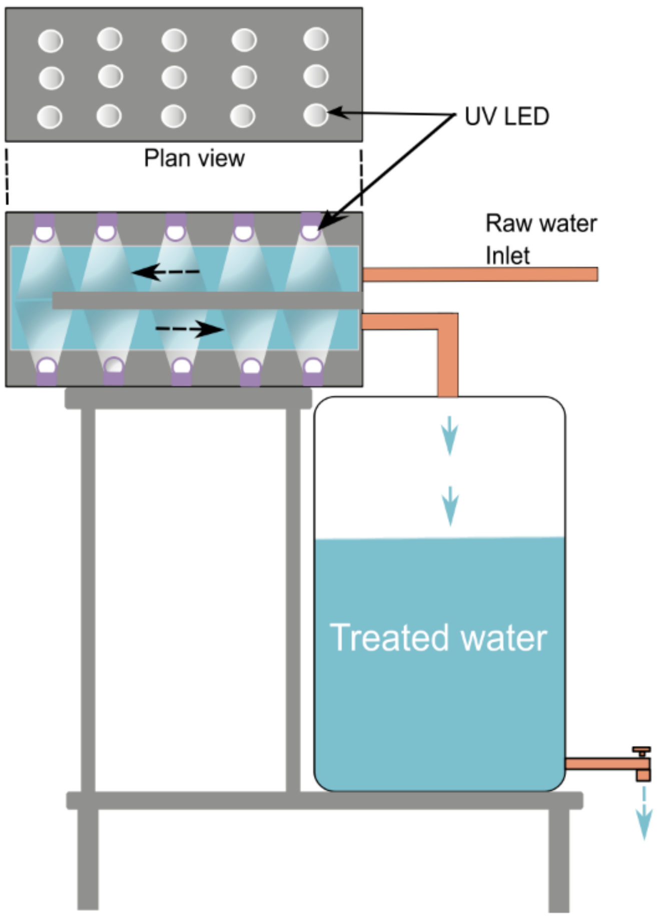

Figure S5. Visual representation of UV disinfection with LEDs. This set-up has water that flows through an array of 30 UV LEDs with 15 LEDs on each side to allow adequate UV transmission for disinfection.

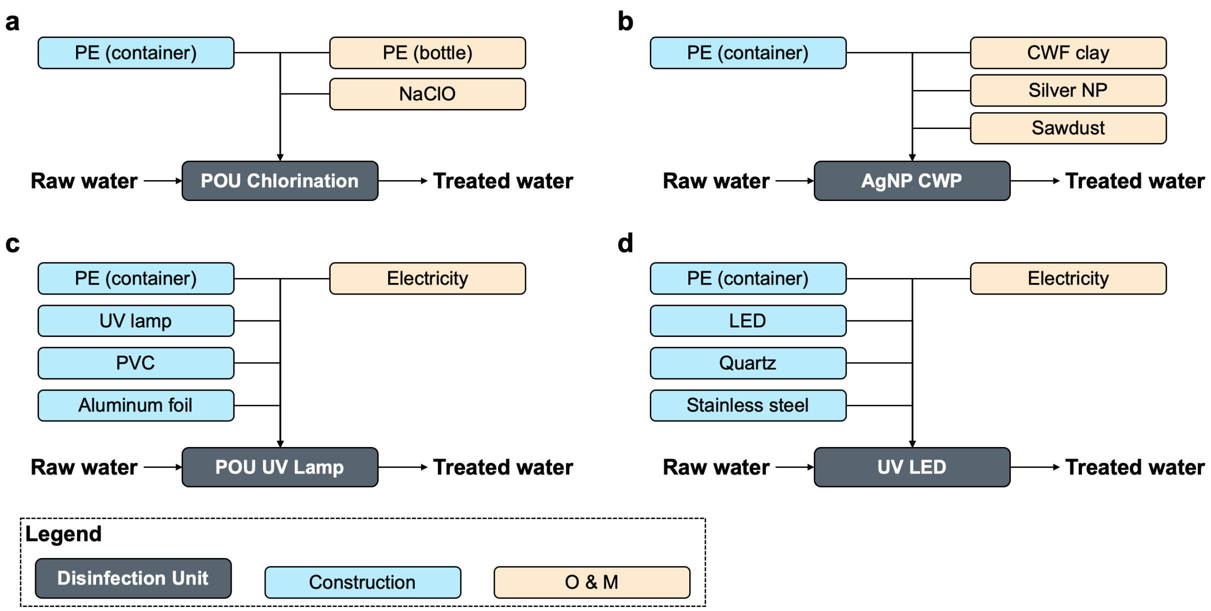

Figure S6. System boundaries and foreground inventory for the four point-of-use (POU) disinfection systems: (a) POU chlorination, (b) silver nanoparticle enabled ceramic water filter (AgNP CWF), (c) POU ultraviolet with mercury lamp (POU UV lamp), and (d) UV with light-emitting diode (UV LED). All inventory items are market activities (i.e., include transportation, if applicable) and are differentiated into construction (blue) or operating and maintenance (O & M).

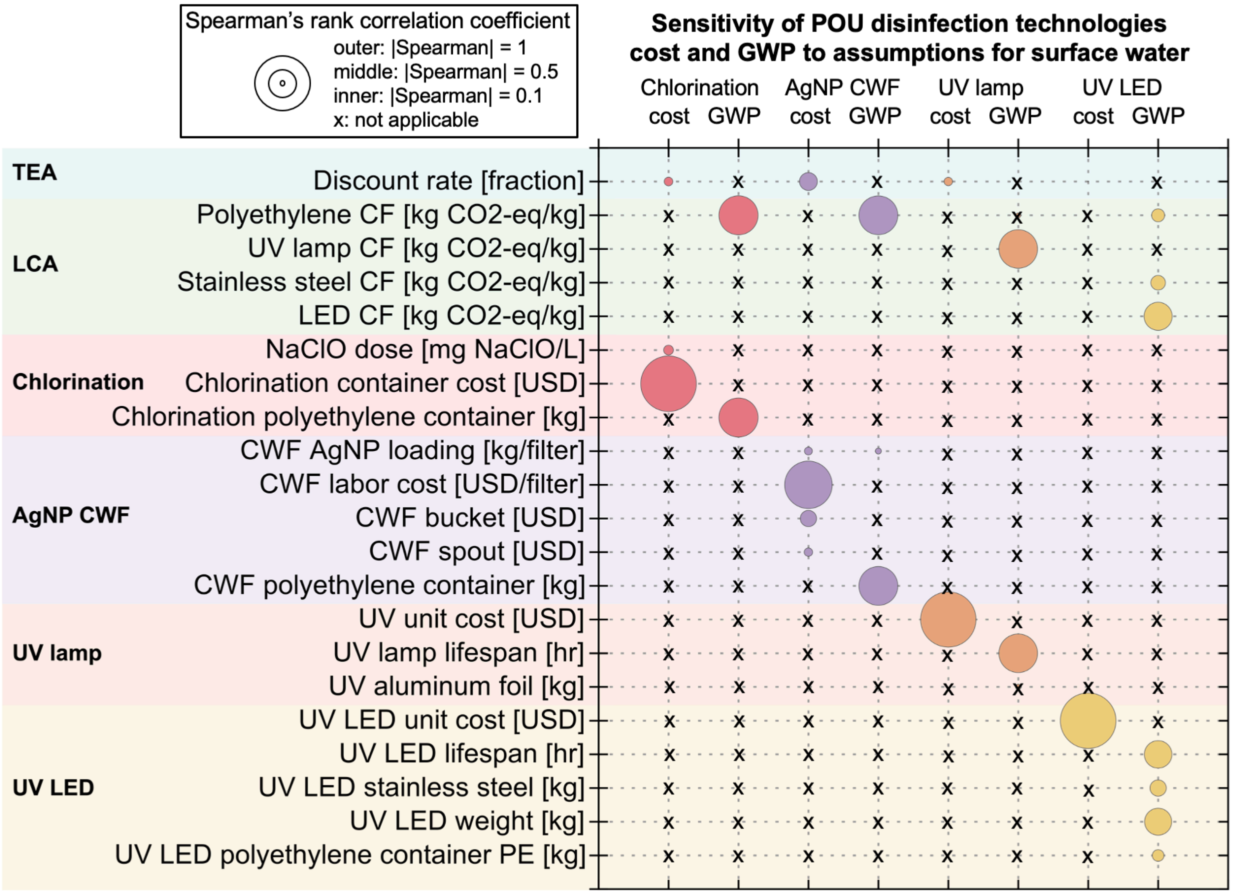

Figure S7. Spearman's rank correlation for net cost and GWP for all POU technologies with groundwater. The key drivers are on the ordinate corresponding with each technology's cost and GWP on the abscissa.

Supporting References

(1) Hussein, M.; Brown, J.; Njee, R. M.; Clasen, T.; Malebo, H. M.; Mbuligwe, S. Point-of-Use Chlorination of Turbid Water: Results from a Field Study in Tanzania. *J. Water Health* **2015**, *13* (2), 544–552. http://dx.doi.org/10.2166/wh.2014.001.

(2) Alibaba. *Plastic Stacking Drums Pails Barrels For Chemical Industry* -. Alibaba.com. https://www.alibaba.com/product-detail/5L-10L-20L-25L-Plastic-Stacking_62585479207.html?spm=a2700.pccps_detail.0.0.3d8f13a046xAC2 (accessed 2023-03-07).

(3) Engineering For Change. *WaterGuard*. Engineering For Change. https://www.engineeringforchange.org/solutions/product/waterguard/ (accessed 2023-04-26).

(4) Aqua-Calc. *Density of Sodium hypochlorite, 14% aqueous solution in 285 units*. https://www.aqua-calc.com/page/density-table/substance/sodium-blank-hypochlorite-coma-and-blank-14-percent-sign--blank-aqueous-blank-solution (accessed 2023-03-07).

(5) Cheswick, R.; Moore, G.; Nocker, A.; Hassard, F.; Jefferson, B.; Jarvis, P. Chlorine Disinfection of Drinking Water Assessed by Flow Cytometry: New Insights. *Environ. Technol. Innov.* **2020**, *19*, 101032. https://doi.org/10.1016/j.eti.2020.101032.

(6) TANKS INTERNATIONAL SRL. *Plastic Approved Jerrycan 20 Litres Volume | Polft20*. https://www.tanksinternational.it/en/category/3/13/PLASTIC-APPROVED-JERRYCAN-20-LITRES-VOLUME/80 (accessed 2023-03-07).

(7) Gerschon. *Gerschon packagings | 150 ml bottle white PE, dispenser twist-lock | purchase online*. https://www.gerschon.de/. https://www.gerschon.de/en/Pharma/Dispenser-Lotion-Pump-oxid/150-ml-bottle-white-PE-dispenser-twist-lock.html (accessed 2023-03-07).

(8) Ren, D.; Colosi, L. M.; Smith, J. A. Evaluating the Sustainability of Ceramic Filters for Point-of-Use Drinking Water Treatment. *Environ. Sci. Technol.* **2013**, *47* (19), 11206–11213. https://doi.org/10.1021/es4026084.

(9) Justman Brush Company. *Economical Wood Handle White Bristle Chip Brush*. https://www.justmanbrush.com/wood-handle-white-bristle-chip-brush/?sku=690256&gclid=EAIaIQobChMIufbJ_Jb9_AIVtf7jBx16WA33EAQYCiABEgJxovD_BwE (accessed 2023-03-07).

(10) Mittelman, A. M.; Lantagne, D. S.; Rayner, J.; Pennell, K. D. Silver Dissolution and Release from Ceramic Water Filters. *Environ. Sci. Technol.* **2015**, *49* (14), 8515–8522. https://doi.org/10.1021/acs.est.5b01428.

(11) Rowles, L. S. Socially Embedded and Sustained Point-of-Use Disinfection : Enhancing Silver Nanoparticle Enabled Ceramic Water Filters with a Navajo Pottery Technique. Thesis, 2020. https://doi.org/10.26153/tsw/13860.

(12) SuSanA. *Best Practice Recommendations for Local Manufacturing of Ceramic Pot Filters for Household Water Treatment*. The Ceramics Manufacturing Working Group. https://www.susana.org/en/knowledge-hub/resources-and-publications/library/details/4124 (accessed 2023-03-07).

(13) Younis, B. A.; Mahoney, L. E.; Yao, S. Field Evaluation of a Novel UV Water Disinfection System for Use in Underserved Rural Communities. *Water Environ. Res. Res. Publ. Water Environ. Fed.* **2018**, *91* (1), 75–82. https://doi.org/10.2175/106143017x15131012188141.

(14) Chatterley, C.; Linden, K. Demonstration and Evaluation of Germicidal UV-LEDs for Point-of-Use Water Disinfection. *J. Water Health* **2010**, *8* (3), 479–486. https://doi.org/10.2166/wh.2010.124.

(15) Jenny, R. M.; Simmons, O. D.; Shatalov, M.; Ducoste, J. J. Modeling a Continuous Flow Ultraviolet Light Emitting Diode Reactor Using Computational Fluid Dynamics. *Chem. Eng. Sci.* **2014**, *116*, 524.

(16) *Audax Electronics Ltda UVC LED Board|CDILED*. Component Distributors, Inc. (CDI). https://led.cdiweb.com/products/detail/80338100100-audax-electronics-ltda/674081/ (accessed 2023-04-26).

(17) Lui, G. Y.; Roser, D.; Corkish, R.; Ashbolt, N. J.; Stuetz, R. Point-of-Use Water Disinfection Using Ultraviolet and Visible Light-Emitting Diodes. *Sci. Total Environ.* **2016**, *553*, 626–635. https://doi.org/10.1016/j.scitotenv.2016.02.039.

(18) Ecoinvent. *Online Access - ecoinvent*. Ecoinvent Database. https://ecoinvent.org/the-ecoinvent-database/online-access/ (accessed 2023-02-13).

(19) Maciel, P. M. F.; Fava, N. de M. N.; Lamon, A. W.; Fernandez-Ibañez, P.; Byrne, J. A.; Sabogal-Paz, L. P. Household Water Purification System Comprising Cartridge Filtration, UVC Disinfection and Chlorination to Treat Turbid Raw Water. *J. Water Process Eng.* **2021**, *43*, 102203. https://doi.org/10.1016/j.jwpe.2021.102203.

(20) Wilhelm, N.; Kaufmann, A.; Blanton, E.; Lantagne, D. Sodium Hypochlorite Dosage for Household and Emergency Water Treatment: Updated Recommendations. *J. Water Health* **2017**, *16* (1), 112–125. https://doi.org/10.2166/wh.2017.012.

(21) Trimmer, J. T.; Lohman, H. A. C.; Byrne, D. M.; Houser, S. A.; Jjuuko, F.; Katende, D.; Banadda, N.; Zerai, A.; Miller, D. C.; Guest, J. S. Navigating Multidimensional Social–Ecological System Trade-Offs across Sanitation Alternatives in an Urban Informal Settlement. *Environ. Sci. Technol.* **2020**, *54* (19), 12641–12653. https://doi.org/10.1021/acs.est.0c03296.

(22) Bwire, G.; Sack, D. A.; Kagirita, A.; Obala, T.; Debes, A. K.; Ram, M.; Komakech, H.; George, C. M.; Orach, C. G. The Quality of Drinking and Domestic Water from the Surface Water Sources (Lakes, Rivers, Irrigation Canals and Ponds) and Springs in Cholera Prone Communities of Uganda: An Analysis of Vital Physicochemical Parameters. *BMC Public Health* **2020**, *20* (1), 1128. https://doi.org/10.1186/s12889-020-09186-3.

(23) Climatescope. *Climatescope 2023 | Uganda*. https://www.global-climatescope.org/markets/ug/ (accessed 2024-02-29).

(24) International Renewable Energy Agency. *Energy Profile - Uganda*. https://www.irena.org/-/media/Files/IRENA/Agency/Statistics/Statistical_Profiles/Africa/Uganda_Africa_RE_SP.pdf (accessed 2024-02-28).

(25) Edokpayi, J. N.; Rogawski, E. T.; Kahler, D. M.; Hill, C. L.; Reynolds, C.; Nyathi, E.; Smith, J. A.; Odiyo, J. O.; Samie, A.; Bessong, P.; Dillingham, R. Challenges to Sustainable Safe Drinking Water: A Case Study of Water Quality and Use across Seasons in Rural Communities in Limpopo Province, South Africa. *Water* **2018**, *10* (2), 159. https://doi.org/10.3390/w10020159.

(26) International Renewable Energy Agency. *Climatescope 2023 | South Africa*. https://www.global-climatescope.org/markets/za/ (accessed 2024-02-29).

(27) International Renewable Energy Agency. *Energy Profile - South Africa*. https://www.irena.org/-/media/Files/IRENA/Agency/Statistics/Statistical_Profiles/Africa/South-Africa_Africa_RE_SP.pdf (accessed 2024-02-28).

(28) Antriyandarti, E.; Barokah, U.; Rahayu, W.; Laia, D. H.; Asami, A. Factors Associated with Food Security of Dryland Farm Households in the Karst Mountains of Gunungkidul Indonesia. *Sustainability* **2023**, *15* (11), 8782. https://doi.org/10.3390/su15118782.

(29) Matthies, K.; Schott, C.; Anggraini, A. K.; Silva, A.; Diedel, R.; Mühlebach, H.; Fuchs, S.; Obst, U.; Brenner-Weiss, G. Drinking Water Treatment for a Rural Karst Region in Indonesia. *Appl. Water Sci.* **2016**, *6* (3), 309–318. https://doi.org/10.1007/s13201-016-0423-2.

(30) Climatescope. *Climatescope 2023 | Indonesia*. https://www.global-climatescope.org/markets/id/ (accessed 2024-02-28).

(31) International Renewable Energy Agency. *Energy Profile - Indonesia*. https://www.irena.org/-/media/Files/IRENA/Agency/Statistics/Statistical_Profiles/Asia/Indonesia_Asia_RE_SP.pdf (accessed 2024-02-28).

(32) Jeco, B. M. F. Y.; Larroder, A. C.; Oguma, K. Technosocial Feasibility Analysis of Solar-Powered UV-LED Water Treatment System in a Remote Island of Guimaras, Philippines. *J. Photonics Energy* **2019**, *9* (4), 043105. https://doi.org/10.1117/1.JPE.9.043105.

(33) Espaldon, A. E.; Yu Jeco-Espaldon, B. M.; Sado, T.; Tenebro, C. P.; Dalisay, D. S.; Oguma, K.; Saludes, J. P. Groundwater Quality Analyses in Off-Grid Tropical Island. *Water Environ. J.* **2022**, *36* (4), 644–655. https://doi.org/10.1111/wej.12804.

(34) Climatescope. *Climatescope 2023 | Philippines*. https://www.global-climatescope.org/markets/ph/ (accessed 2024-02-28).

(35) International Renewable Energy Agency. *Energy Profile - Philippines*. https://www.irena.org/-/media/Files/IRENA/Agency/Statistics/Statistical_Profiles/Asia/Philippines_Asia_RE_SP.pdf (accessed 2024-02-28).

(36) Rowles, L. S.; Whittaker, T.; Ward, P. M.; Araiza, I.; Kirisits, M. J.; Lawler, D. F.; Saleh, N. B. A Structural Equation Model to Decipher Relationships among Water, Sanitation, and Health in Colonias-Type Unincorporated Communities. *Environ. Sci. Technol.* **2020**, *54* (24), 16017–16027. https://doi.org/10.1021/acs.est.0c05355.

(37) Rowles III, L. S.; Hossain, A. I.; Ramirez, I.; Durst, N. J.; Ward, P. M.; Kirisits, M. J.; Araiza, I.; Lawler, D. F.; Saleh, N. B. Seasonal Contamination of Well-Water in Flood-Prone Colonias and Other Unincorporated U.S. Communities. *Sci. Total Environ.* **2020**, *740*, 140111. https://doi.org/10.1016/j.scitotenv.2020.140111.
(38) United States Environmental Protection Agency. *eGRID Data Explorer*. https://www.epa.gov/egrid/data-explorer (accessed 2024-02-29).
(39) Rowles, L. S.; Tso, D.; Dolocan, A.; Kirisits, M. J.; Lawler, D. F.; Saleh, N. B. Integrating Navajo Pottery Techniques To Improve Silver Nanoparticle-Enabled Ceramic Water Filters for Disinfection. *Environ. Sci. Technol.* **2023**, *57* (44), 17132–17143. https://doi.org/10.1021/acs.est.3c03462.
(40) Roy, M. A.; Arnaud, J. M.; Jasmin, P. M.; Hamner, S.; Hasan, N. A.; Colwell, R. R.; Ford, T. E. A Metagenomic Approach to Evaluating Surface Water Quality in Haiti. *Int. J. Environ. Res. Public. Health* **2018**, *15* (10), 2211. https://doi.org/10.3390/ijerph15102211.
(41) International Renewable Energy Agency. *Climatescope 2023 | Haiti*. https://www.global-climatescope.org/markets/ht/ (accessed 2024-02-29).
(42) International Renewable Energy Agency. *Energy Profile - Haiti*. https://www.irena.org/-/media/Files/IRENA/Agency/Statistics/Statistical_Profiles/Central-America-and-the-Caribbean/Haiti_Central-America-and-the-Caribbean_RE_SP.pdf (accessed 2024-02-28).
(43) Peláez, M. A. Z. Implementing a UV Disinfection System in a Low-Income Area of Bolivia, South America, University of Alberta, 2011. https://doi.org/10.7939/R3VM7G.
(44) Climatescope. *Climatescope 2023 | Bolivia*. https://www.global-climatescope.org/markets/bo/ (accessed 2024-02-28).
(45) International Renewable Energy Agency. *Energy Profile - Bolivia (Plurinational State of)*. https://www.irena.org/-/media/Files/IRENA/Agency/Statistics/Statistical_Profiles/South-America/Bolivia-Plurinational-State-of_South-America_RE_SP.pdf (accessed 2024-02-28).
(46) Global Data Lab. *Average household size*. https://globaldatalab.org/areadata/table/hhsize/COL/ (accessed 2024-02-28).
(47) Botero, L.; Galeano, L.; Montoya, L. J.; Machado, A.; Byrne, J. A.; Fernandez-Ibañez, P.; Hincapié, M. *Aeromonas Hydrophila* in Surface Water and Their Removal Using a POU Technology for Drinking in Rural Communities. *Environ. Adv.* **2023**, *13*, 100425. https://doi.org/10.1016/j.envadv.2023.100425.
(48) *Climatescope 2023 | Colombia*. https://www.global-climatescope.org/markets/co/ (accessed 2024-02-28).
(49) International Renewable Energy Agency. *Energy Profile - Colombia*. https://www.irena.org/-/media/Files/IRENA/Agency/Statistics/Statistical_Profiles/South%20America/Colombia_South%20America_RE_SP.pdf (accessed 2024-02-28).
(50) Rowles, L. S.; Alcalde, R.; Bogolasky, F.; Kum, S.; Diaz-Arriaga, F. A.; Ayres, C.; Mikelonis, A. M.; Toledo-Flores, L. J.; Alonso-Gutiérrez, M. G.; Pérez-Flores, M. E.; Lawler, D. F.; Ward, P. M.; Lopez-Cruz, J. Y.; Saleh, N. B. Perceived versus Actual Water Quality: Community Studies in Rural Oaxaca, Mexico. *Sci. Total Environ.* **2018**, *622–623*, 626–634. https://doi.org/10.1016/j.scitotenv.2017.11.309.
(51) Climatescope. *Climatescope 2023 | Mexico*. https://www.global-climatescope.org/markets/mx/ (accessed 2024-02-29).
(52) International Renewable Energy Agency. *Energy Profile - Mexico*. https://www.irena.org/-/media/Files/IRENA/Agency/Statistics/Statistical_Profiles/North%20America/Mexico_North%20America_RE_SP.pdf (accessed 2024-02-28).
